# PICALM modulates autophagy activity and tau accumulation

**DOI:** 10.1038/ncomms5998

**Published:** 2014-09-22

**Authors:** Kevin Moreau, Angeleen Fleming, Sara Imarisio, Ana Lopez Ramirez, Jacob L. Mercer, Maria Jimenez-Sanchez, Carla F. Bento, Claudia Puri, Eszter Zavodszky, Farah Siddiqi, Catherine P. Lavau, Maureen Betton, Cahir J. O’Kane, Daniel S. Wechsler, David C. Rubinsztein

**Affiliations:** 1 Department of Medical Genetics, University of Cambridge, Cambridge Institute for Medical Research, Addenbrooke’s Hospital, Cambridge Biomedical Campus, Hills Road, Cambridge CB2 0XY, UK; 2 Department of Physiology, Development and Neuroscience, University of Cambridge, Downing Street, Cambridge CB2 3EG, UK; 3 Department of Genetics, University of Cambridge, Downing Street, Cambridge CB2 3EG, UK; 4 Department of Pediatrics, Duke University Medical Center, Durham, North Carolina 27710, USA; 5 Department of Pharmacology and Cancer Biology, Duke University Medical Center, Durham, North Carolina 27710, USA

## Abstract

Genome-wide association studies have identified several loci associated with Alzheimer’s disease (AD), including proteins involved in endocytic trafficking such as PICALM/CALM (phosphatidylinositol binding clathrin assembly protein). It is unclear how these loci may contribute to AD pathology. Here we show that CALM modulates autophagy and alters clearance of tau, a protein which is a known autophagy substrate and which is causatively linked to AD, both *in vitro* and *in vivo*. Furthermore, altered CALM expression exacerbates tau-mediated toxicity in zebrafish transgenic models. CALM influences autophagy by regulating the endocytosis of SNAREs, such as VAMP2, VAMP3 and VAMP8, which have diverse effects on different stages of the autophagy pathway, from autophagosome formation to autophagosome degradation. This study suggests that the AD genetic risk factor CALM modulates autophagy, and this may affect disease in a number of ways including modulation of tau turnover.

## Abstract (web summary)

The protein PICALM/CALM is implicated in Alzheimer’s disease (AD) pathology, but it is unclear how. In this study, the authors show that CALM regulates clearance of the protein tau, which is also implicated in AD pathology, by facilitating endocytosis-dependent autophagy.

## Graphical abstract

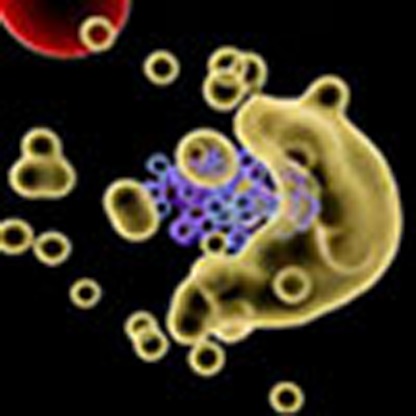

Alzheimer’s disease (AD) is the most common dementia. Its brain pathology is characterized by the accumulation and aggregation of extracellular β-amyloid plaques and intracytoplasmic tau, the latter correlating better with disease progression^1^. (Macro)autophagy is a critical clearance pathway for organelles and long-lived proteins, including intracytoplasmic aggregate-prone proteins that cause many neurodegenerative diseases, such as huntingtin in Huntington’s disease and tau in AD (reviewed in ref. 2). Furthermore, autophagy protects cells against proapoptotic insults by reducing caspase activation^3^. One of the hallmarks of AD pathology is an excess of autophagosomes in affected neurons, suggesting that there may be defects in autophagosome removal^4^. However, autophagy may be perturbed at multiple steps in AD, including additional effects at the levels of autophagosome biogenesis (reviewed in ref. 5), and its upregulation has benefits in various AD animal models^6^,^7^,^8^.

Autophagosomes are double-membraned structures that engulf portions of cytoplasm and ultimately fuse with lysosomes, where their contents are degraded. Autophagy begins with the formation of cup-shaped structures called phagophores, whose edges extend and fuse to form autophagosomes^9^,^10^. The ATG5-ATG12/ATG16L1 complex regulates the initiation of phagophore formation, while phosphatidylethanolamine-conjugated ATG8/LC3 (LC3-II) mediates the elongation and fusion of the phagophore edges to form autophagosomes^9^,^10^. The ATG5-ATG12/ATG16L1 complex decorates the phagophore and dissociates after completion of autophagosome formation, while LC3-II is localized to both the phagophore and fully formed autophagosomes, and its levels correlate with autophagosome number.

Autophagosomes may derive membrane from diverse sources, including plasma membrane, recycling endosomes, Golgi, endoplasmic reticulum and mitochondria^11^,^12^,^13^,^14^,^15^,^16^,^17^,^18^. Clathrin-mediated endocytosis regulates autophagy by enabling membrane delivery to ATG5/ATG12/ATG16L1-positive phagophore precursor vesicles (LC3-negative), which mature to form phagophores (ATG16L1-positive and LC3-positive), and subsequently autophagosomes (ATG16L1-negative and LC3-positive)^12^. The ATG16L1-positive/LC3-negative phagophore precursors undergo homotypic fusion events that increase their size and enhance their ability to acquire LC3-II. These fusion events are mediated by soluble NSF attachment protein receptors (SNAREs), including VAMP7, syntaxin 7, syntaxin 8 and VTI1B, although our data suggested that additional SNAREs were likely to be mediators of this process as well^16^. Maturation of the ATG16L1-positive precursors also requires VAMP3-mediated fusion with ATG9-positive vesicles in recycling endosomes^18^. Interestingly, VAMP3 depletion does not affect ATG16L1 homotypic fusion^18^. SNAREs also regulate autophagosome degradation. For instance, VAMP8 on lysosomes associates with partner SNAREs such as VTI1B on autophagosomes to enable fusion of these compartments^19^.

Recent genome-wide association studies (GWAS) have identified several loci associated with AD risk^20^,^21^. It is unclear how most of these have an impact on the genesis of AD. Although no direct autophagy genes were identified, these loci encode a number of genes involved in endocytic trafficking. One of these, *PICALM* (also known as *CALM*), has been validated in independent studies^22^,^23^,^24^. Phosphatidylinositol-binding clathrin assembly protein (PICALM/CALM) appears to be abnormally cleaved in AD samples and levels of uncleaved full-length CALM are significantly decreased in AD brains, suggesting that decreased CALM function may be relevant in this disease^25^. CALM encodes a clathrin adaptor that regulates the orderly progression of coated bud formation at the plasma membrane. It directly interacts with and thereby regulates the endocytosis of SNAREs, such as VAMP2, VAMP3 and VAMP8 (refs 26, 27). As endocytosis and SNAREs both regulate autophagy, we tested the role of CALM in autophagy and its relevance in a model of neurodegeneration *in vivo*. We found that CALM regulates autophagy and clearance of tau, an autophagy substrate, thus providing a potential mechanism by which CALM levels might influence the severity of AD.

## Results

### CALM modulates autophagy

**Figure 1:**
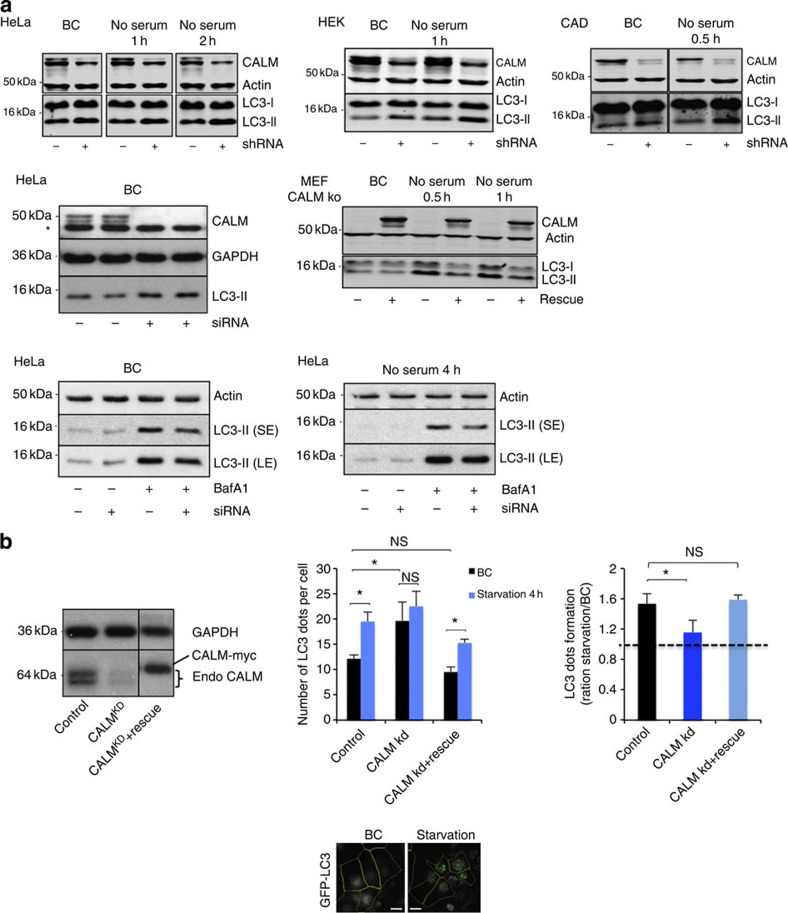
CALM modulates autophagy. (**a**) Western blot analysis of CALM, actin, GAPDH and LC3-II in several cell lines (HeLa, HEK, CAD and murine embryonic fibroblast (MEF)) where CALM was knocked down using shRNA or siRNA, as indicated, or knockout with rescue experiment. In all experiments in this paper, we used a scramble siRNA or a luciferase shRNA as controls. The cells were starved in Hanks balanced salt solution (HBSS) and treated with Baf A1 as indicated. (BC, basal conditions; SE, short exposure; LE, longer exposure). Quantification of LC3-II/actin or GAPDH ratio is shown in Supplementary Fig. 1A. *Not specific. (**b**) LC3 dot counting in CALM knockdown cells. CALM was knocked down in HeLa cells expressing GFP-LC3 and with or without an siRNA-resistant form of wild-type CALM (rescue), as indicated. The cells were starved in HBSS for 4 h, fixed and subjected to microscopy to score the number of LC3 dots per cell. The knockdown efficiency and the level of the siRNA-resistant form of CALM are shown on the left on the western blotting. The number of LC3 dots per cell (shown as mean ±s.d.) is shown on the graph for each condition (*n*≥300 cells per condition; BC, basal conditions). The ratio of the number of LC3 dots per cell between starvation and basal conditions is shown on the right (*P<0.01; NS, not significant, two-tailed *t*-test). Pictures obtained from automated microscope are shown. Scale bars, 20 μm.

We tested the role of CALM in autophagy and observed increased LC3-II levels in HeLa, HEK (human embryonic kidney), CAD (catecholaminergic cell line) and primary neurons, where CALM was knocked down using short hairpin RNA (shRNA) or small interfering RNA (siRNA) under basal conditions (BCs), and in HeLa, HEK and CAD cells exposed to serum starvation, an autophagy stimulus (Fig. 1a and Supplementary Fig. 1a,b)^28^. The increase in LC3-II levels due to CALM depletion was rescued in CALM-null mouse embryonic fibroblasts by reintroducing CALM using a viral system (Fig. 1a). These experiments suggest that CALM deficiency influences autophagy, either by stimulating autophagosome formation or by inhibiting their degradation, both of which increase LC3-II levels. To discriminate between these possibilities, LC3-II levels was measured in the presence of saturating concentrations of bafilomycin A1 (Baf A1), a potent inhibitor of the vacuolar H+ ATPase that interferes with the degradation of autophagosomes/LC3-II by preventing lysosomal acidification^28^. CALM knockdown decreased LC3-II levels in the presence of Baf A1 in BCs in HeLa cells and primary neurons, suggesting reduced autophagosome formation (Fig. 1a and Supplementary Fig. 1a,b). This effect remained robust when autophagy was stimulated by starvation, where CALM knockdown decreased the amount of LC3-II in the presence of Baf A1 by about 40% in HeLa cells (Fig. 1a and Supplementary Fig. 1a). This is similar to the effects seen when we knock down key autophagy genes^16^ as well as genes involved in clathrin-mediated endocytosis^12^. The decreased LC3-II levels in Baf A1-treated conditions and the increase of LC3-II in Baf A1-untreated conditions suggest that CALM modulates both autophagosome synthesis and autophagosome degradation. To further characterize the role of CALM in autophagy, we used another assay to measure autophagy. LC3 dot numbers (autophagosomes) increased in CALM knockdown cells compared with control in BCs (Fig. 1b), which, if viewed in isolation, could be either due to an increase in autophagosome formation or a defect in their clearance. Serum starvation increased the number of LC3 dots per cell in control cells, but caused no further increase above nutrient-replete medium in CALM knockdown cells (Fig. 1b), suggesting that CALM indeed affects both autophagosome biosynthesis and autophagosome clearance, consistent with the LC3 western blotting data above. We confirmed that an siRNA-resistant myc-CALM construct could rescue the autophagy defect caused by CALM siRNA (Fig. 1b). Thus, CALM knockdown appears to regulate both autophagosome formation and degradation. These have opposing effects on basal LC3-II levels, but CALM knockdown has clear effects on autophagosome biogenesis when we clamp LC3 degradation with Baf A1, or score autophagic vesicles in starved cells.

CALM overexpression, which affects clathrin-mediated traffic when expressed at high levels^29^, also decreased autophagosome formation, as assessed by LC3-II levels (with or without Baf A1) (Supplementary Fig. 1c). The inhibition of autophagosome formation in CALM-overexpressing cells was associated with decreased transferrin uptake (Supplementary Fig. 1d), suggesting decreased endocytosis, but did not affect endocytosis of various SNAREs (VAMP2, VAMP3 and VAMP8; Supplementary Fig. 1f). A CALM mutant that includes the ANTH domain but not the clathrin-interacting domain (and which did not inhibit transferrin uptake, Supplementary Fig. 1d) did not decrease autophagy, as had similar LC3-II levels to control cells (Supplementary Fig. 1c). The role of CALM in transferrin internalization is controversial^26^,^29^,^30^. Although CALM seems to influence transferrin uptake *in vivo* in CALM-knockout mice^31^, it is not so clear *in vitro*^30^,^32^. We observed no defect in transferrin internalization with CALM knockdown, while we observed a clear defect in epidermal growth factor uptake (Supplementary Fig. 1e). These data raise the question of whether CALM regulates only endocytosis of specific substrates, rather than bulk clathrin-mediated endocytosis^33^. Our data suggest that CALM knockdown, which affects SNARE endocytosis^32^ (confirmed later in this paper), decreases both autophagosome formation and autophagosome clearance. On the other hand, CALM overexpression does not affect VAMP2, VAMP3 or VAMP8 endocytosis and predominantly inhibits autophagosome biogenesis without affecting autophagosome clearance.

**Figure 2:**
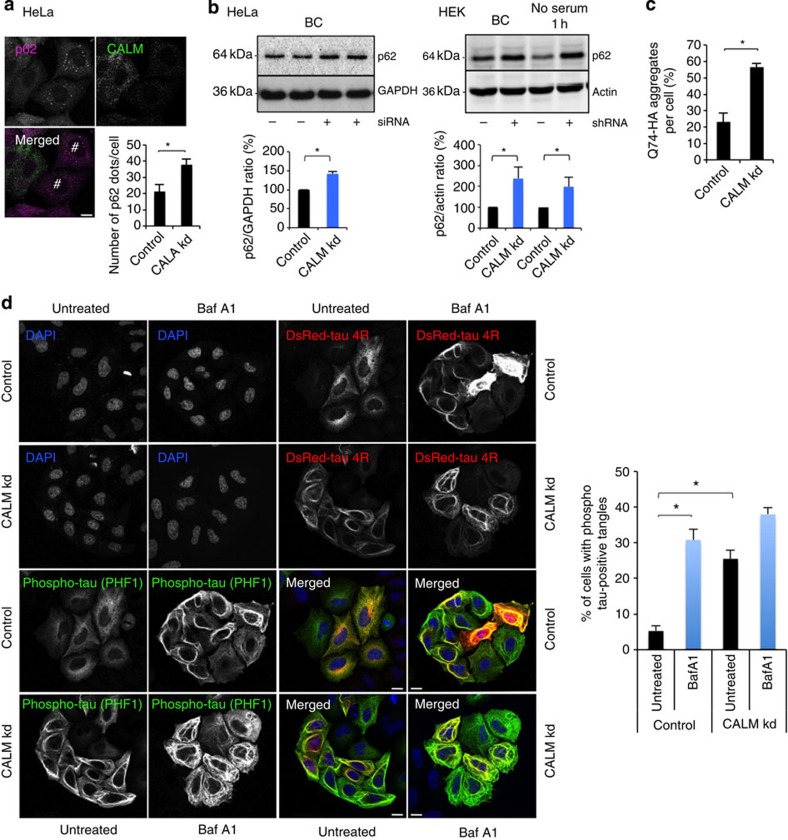
CALM modulates autophagy substrate clearance *in vitro*. (**a**) p62 vesicle formation in CALM knockdown HeLa cells. Confocal pictures are shown. #CALM-downregulated cells where p62 vesicles accumulate. Data are representative of three independent experiments and shown as mean ±s.e.m. (*n*≥500 cells; *P<0.01; two-tailed *t*-test). Scale bars, 5 μm. (**b**) Western blot analysis of p62, actin and GAPDH in HeLa cells and HEK cells (basal conditions, BC, or without serum for 1 h) where CALM was knocked down using shRNA or siRNA, as indicated. Data are mean ±s.d (*n*=3 experiments for HeLa cells and HEK cells; *P<0.05; two-tailed *t*-test). (**c**) Percentage of Q74-expressing cells with aggregates in CALM knockdown HeLa cells. Data depict one representative experiments performed in triplicate, out of three independent experiments and shown as mean ±s.d. (*P<0.05; two-tailed *t*-test). (**d**) Tau-positive tangle formation in CALM knockdown cells. HeLa cells transiently expressing DsRed-tau 4R were treated with Baf A1 for 4 h as indicated. Cells were fixed and analysed by confocal microscopy after immnunostaining for phosphorylated tau using PHF1 antibody (green). Data represent the number of cells with phosphorylated tau-positive tangles as mean ±s.e.m. (*n*=3 experiments; *P<0.01; two-tailed *t*-test). Scale bars, 5 μm.

The inhibition of autophagosome formation and degradation when CALM was downregulated was associated with the accumulation of autophagic substrates, as measured by the number of endogenous p62 dots per cell, p62 levels by western blotting or the number of mutant huntingtin (Q74) aggregates per cell (huntingtin mutant with 74 glutamine repeats) (Fig. 2ac). Both of these proteins aggregate when autophagy is impaired and the number of mutant huntingtin aggregates correlates linearly with protein abundance under normal conditions^34^. We also observed an accumulation of AD associated tau, another autophagy substrate, and its phosphorylated form (using PHF1 antibody) by immunofluorescence in structures resembling tau tangles (a hallmark of AD) in cells expressing DsRed-tau 4R when autophagy was inhibited by Baf A1 or when CALM was knocked down (Fig. 2d).

### CALM regulates autophagic precursor formation

To understand how CALM affects autophagy, we assessed the localization of endogenous CALM on autophagic structures associated with endogenous ATG12 and LC3. First, co-localization between CALM and AP2 confirmed that our CALM immunostaining was specific because both proteins are known to be localized in clathrin-coated vesicles (Supplementary Fig. 2a). Furthermore, we observed CALM on ATG12 vesicles that did not contain LC3 (phagophore precursors), as well as on vesicles positive for both ATG12 and LC3 (phagophores), suggesting that autophagic precursors acquire CALM at an early stage of autophagosome formation (Supplementary Fig. 2a). The presence of CALM on ATG12 vesicles was increased in cells expressing a mutant form of ATG4B (ATG4B C74A) that impairs the progression from phagophores to autophagosomes^35^, further supporting the presence of CALM on early autophagic structures (Supplementary Fig. 2a). We confirmed the localization of CALM on autophagic precursors by immuno-electron microscopy, where CALM co-localized with ATG16L1 (Supplementary Fig. 2b).

**Figure 3:**
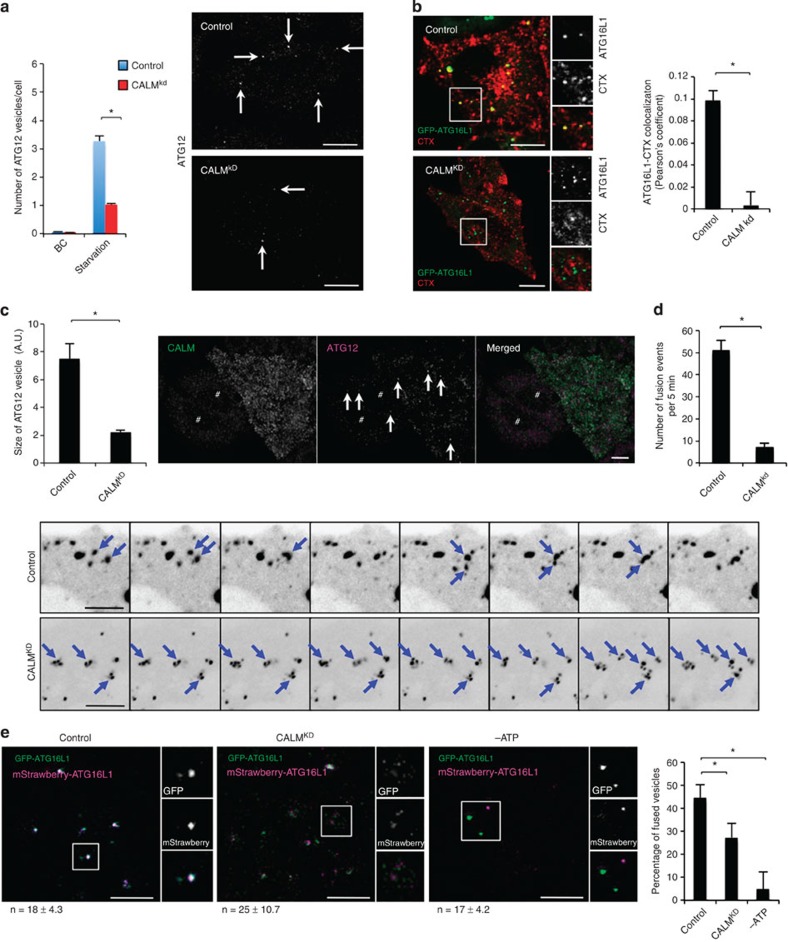
CALM regulates the formation and maturation of autophagic precursors. (**a**) Formation of endogenous ATG12 vesicles in CALM knockdown HeLa cells in basal (BC) and serum starvation conditions. Confocal pictures are shown with arrows indicating ATG12 vesicles in starvation conditions. Data are from one representative experiment, out of three independent experiments. Data shown as mean ±s.e.m. (*n*≥500 cells; *P<0.01; two-tailed *t*-test). Scale bars, 5 μm. (**b**) Co-localization between GFP-ATG16L1 and internalized cholera toxin subunit B conjugated with Alexa555 (CTX; 20 min) in CALM knockdown HeLa cells. Confocal pictures are presented with magnified areas showing the co-localization between ATG16L1 and CTX in greater detail. Scale bars, 5 μm. The Pearson’s coefficient between ATG16L1 and cholera toxin is shown. Data are representative of three independent experiments and shown as mean ±s.d. (*n*≥20 cells; *P<0.05; two-tailed *t*-test). (**c**) Size of endogenous ATG12 vesicles in CALM knockdown HeLa cells in starvation conditions. Confocal pictures are shown with arrows indicating ATG12 vesicles and # indicating CALM knockdown cells. Data are representative of three independent experiments and shown as mean ±s.d. (*n*≥100 vesicles; *P<0.05; two-tailed *t*-test). (AU, arbitrary unit). Scale bars, 5 μm. (**d**) Live-cell imaging of ATG16L1-GFP in CALM knockdown HeLa cells. Confocal pictures from various time points of a 5-min movie are shown in inverted grey style. Arrows indicate ATG16L1 vesicles. The number of fusion events per 5 min is shown. Data are representative of five movies and shown as mean ±s.d. (*P<0.05; two-tailed *t*-test). Scale bars, 5 μm. (**e**) *In vitro* fusion assay of post-nuclear supernatant from HeLa cells expressing either GFP-ATG16L1 or mStrawberry-ATG16L1 in control and CALM knockdown conditions. Confocal pictures are shown where ATG16L1-mStrawberry signal is shown in purple to enable better visualization. Fused vesicles appear in white. The ATP-negative condition, which prevents SNARE-dependent fusion, is also shown as a control for the reaction. Magnified areas are shown to allow visualization of the vesicles. The percentage of fused vesicles is represented. *n*=numbers of vesicles scored per field (a minimum of five fields were analysed per condition). Data are representative of two independent experiments and shown as mean ±s.d. (*n*≥100 vesicles). Scale bars, 5 μm. (*P<0.05; two-tailed *t*-test).

Given the presence of CALM on autophagic precursors, we studied the effects of CALM depletion on the formation of these precursors. CALM knockdown reduced the numbers of endogenous ATG12 vesicles or GFP-ATG16L1 vesicles per cell and the association of plasma membrane with autophagic precursors, as measured by the co-localization of GFP-ATG16L1 and internalized cholera toxin (used as a plasma membrane tracer) (Fig. 3a,b and Supplementary Fig. 2c)^12^. This phenocopied what we observed when we impaired clathrin-mediated endocytosis by knocking down either clathrin heavy chain or AP2 (ref. 12). These data suggest that one mechanism for the effect of CALM deficiency on autophagosome biogenesis is by reducing the levels of ATG5/ATG12/ATG16L1-positive phagophore precursors, which correlates with a subsequent defect in autophagosome formation^12^. However, we noted that the size of the ATG12 vesicles or ATG16L1-GFP vesicles was also smaller in CALM knockdown cells (Fig. 3c and Supplementary Fig. 2d). We therefore investigated the impact of reduced CALM levels on ATG16L1 homotypic fusion. Using live imaging, we confirmed fewer homotypic fusion events involving such phagophore precursors in CALM knockdown cells (Fig. 3d and Supplementary Movies 1 and 2). However, this assay may be misleading by itself, if one starts with fewer vesicles. Another way to measure the rate of fusion events between ATG16L1 vesicles is to perform an *in vitro* fusion assay between such vesicles derived from cells expressing either green fluorescent protein (GFP)- or mStrawberry-tagged ATG16L1, as previously described^16^. In this assay, the fusions are expressed as a function of the number of vesicles. As we also observed fewer fusion events using ATG16L1 vesicles from CALM knockdown cells (Fig. 3e), it is possible that CALM regulates the levels of proteins that are required for these fusion events, such as SNAREs^16^. The absence of ATP in the system abolished the fusion between two sets of vesicles, consistent with a role for SNAREs in the process (Fig. 3e)^16^.

We recently described another step required for autophagosome formation that may be relevant to CALM. ATG16L1-positive precursors undergo heterotypic fusion with ATG9A-positive vesicles and this is important for subsequent maturation of these autophagic precursors into autophagosomes^18^. Therefore, we assessed the interaction between ATG16L1 and ATG9A vesicles using confocal microscopy. We observed a decrease in ATG16L1-ATG9A co-localization in CALM knockdown cells (Supplementary Fig. 2e), suggesting a level of regulation at early steps of autophagosome biogenesis by CALM. Moreover, we observed a decrease in LAMP1-LC3 co-localization in CALM knockdown cells (Supplementary Fig. 2f), suggesting a defect in autophagosome/lysosome fusion, which could explain the increase in LC3-II levels seen on western blotting (as shown in Fig. 1a).

### CALM-dependent SNARE endocytosis is required for autophagy

**Figure 4:**
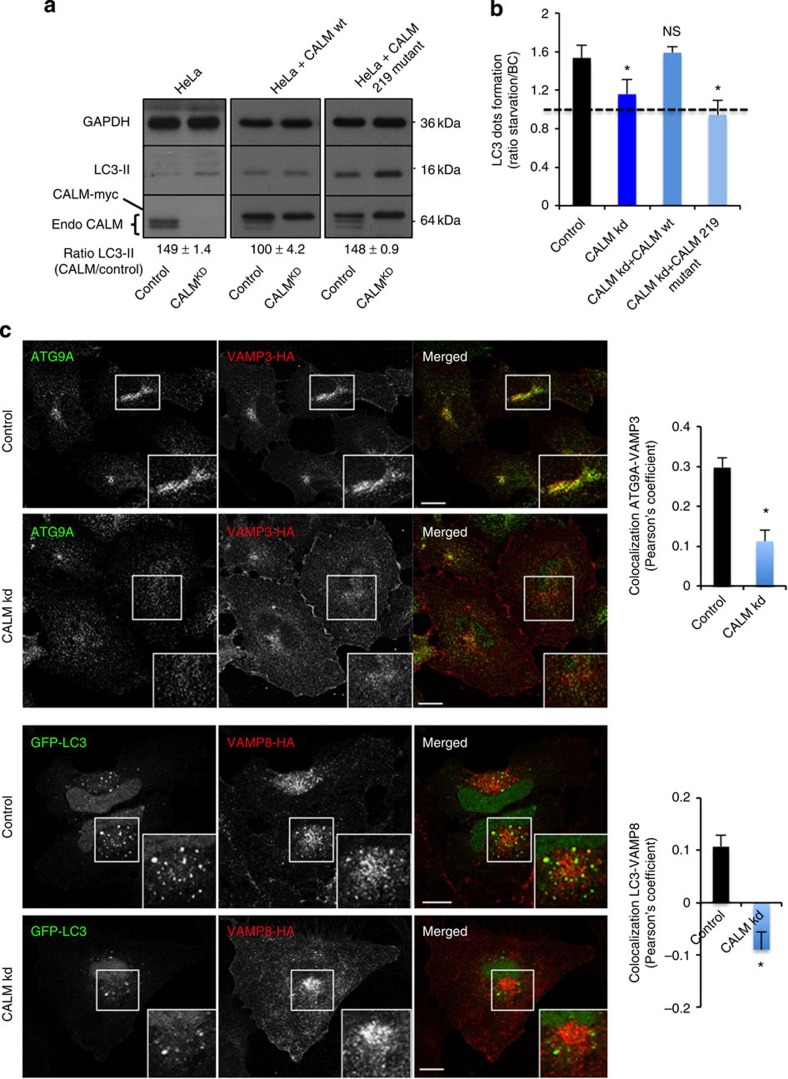
CALM affects autophagy via SNARE endocytosis. (**a**) LC3 levels in CALM knockdown cells. CALM was knocked down in cells expressing or not expressing an siRNA-resistant form of CALM wild type (wt) or CALM 219 mutant, as indicated. The cells were lysed and subjected to western blotting. The knockdown efficiency, LC3-II levels and the level of the siRNA-resistant form of CALM are shown on the western blotting. (**b**) LC3 dots counting in CALM knockdown cells. CALM was knocked down in cells expressing GFP-LC3 with or without an siRNA-resistant form of CALM wild type (wt) or CALM mutant (219 mutant) as indicated. The cells were kept in full medium or starved in Hanks balanced salt solution (HBSS) for 4 h, fixed and subjected to microscopy to score the number of LC3 dots per cell. The ratio of the number of LC3 dots per cell (shown as mean ±s.d.) between starvation and basal conditions is shown on the graph (*P<0.01; NS, not significant, two-tailed *t*-test; *n*≥300 cells per condition; BC, basal conditions). (**c**) Co-localization between ATG9A and VAMP3-HA or GFP-LC3 and VAMP8-HA in control and CALM knockdown HeLa cells stably expressing VAMP3-HA or VAMP8-HA. Confocal pictures are shown with magnified areas showing co-localization between ATG9A and VAMP3 or GFP-LC3 and VAMP8 in control cells and no co-localization in CALM knockdown cells. Quantification of ATG9A-VAMP3 or GFP-LC3-VAMP8 co-localization is shown on the graph as a Pearson’s coefficient (data are mean ±s.d.; *P<0.05; two-tailed *t*-test). Scale bars, 5 μm.

Our observations that CALM knockdown affects both autophagosome formation associated with defects at both homotypic and heterotypic fusion steps, as well as impaired autophagosome degradation associated with defective autophagosome/lysosome fusion is consistent with defects in the functions of diverse SNAREs. CALM regulates the endocytosis of many SNAREs, such as VAMP2, VAMP3 and VAMP8 (ref. 27). VAMP3 regulates heterotypic fusion between ATG16L1 and ATG9A precursors, and is associated with ATG9A-containing vesicles emanating from the plasma membrane^18^, while VAMP8 has been shown to regulate autophagosome–lysosome fusion^19^, and nothing is known about the role of VAMP2 in autophagy. To test the possibility that CALM modulates autophagy via its role in SNARE endocytosis, we used a previously characterized siRNA-resistant form of CALM mutated in the SNARE binding site (CALM 219 mutant)^32^. We observed an increase of LC3-II in CALM knockdown cells, as seen previously (Fig. 4a). Although the expression of siRNA-resistant wild-type CALM in the knockdown cells was able to reduce the levels LC3-II to control levels, an siRNA-resistant CALM 219 mutant (that rescued EGF uptake but not SNAREs internalization) was not able to rescue the LC3-II levels, as seen by the increase of LC3-II compared with the control (Fig. 4a and Supplementary Fig. 3a). The inability of this CALM mutant to rescue CALM knockdown was also observed by microscopy, when we assessed the number of LC3 dots in basal or starvation conditions (Fig. 4b). Although the wild-type CALM-expressing cells were able to produce new autophagosomes on starvation (as seen by the ratio of the number of LC3 dots per cell between starvation and BCs), the mutant CALM was not able to do so (Fig. 4b). These data suggest that the effects of CALM knockdown on autophagy can be attributed to the role of CALM in regulating endocytosis of SNAREs, such as VAMP2, VAMP3 and VAMP8. Consistent with this hypothesis, we observed decreased ATG9A/VAMP3 co-localization (regulated by VAMP3 (ref. 18)) and LC3/VAMP8 co-localization (regulated by VAMP8 (ref. 19)) in CALM knockdown cells, where these two SNAREs accumulated at the plasma membrane (Fig. 4c). Consistent with a role for VAMP3 in autophagosome biogenesis, we observed decreased LC3 vesicles and elevated p62 dots with VAMP3 knockdown (Supplementary Fig. 3b,d). Similarly, VAMP8 knockdown increased the numbers of p62 dots and elevated LC3-II levels in the absence of Baf A1, while having minimal effects in the presence of Baf A1, consistent with previous data showing that this would impair autophagosome degradation^19^ (Supplementary Fig. 3b–d).

### VAMP2 endocytosis is required for autophagosome formation

**Figure 5:**
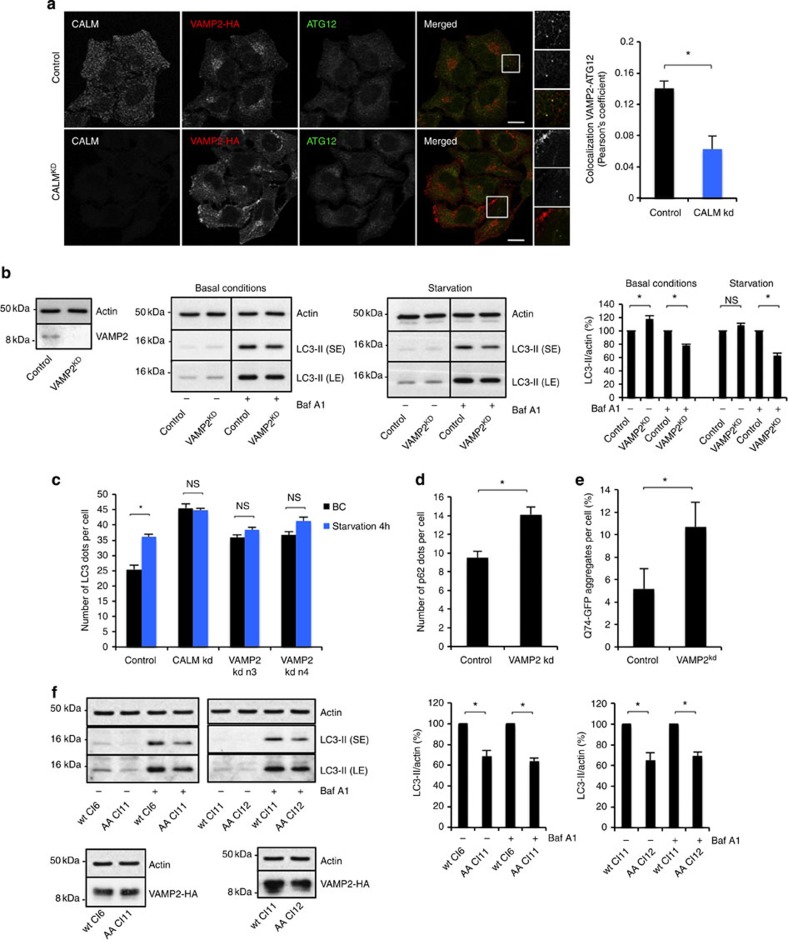
VAMP2 regulates autophagosome formation. (**a**) Co-localization between ATG12 and VAMP2-HA in control and CALM knockdown HeLa cells stably expressing VAMP2-HA. Confocal pictures are shown with magnified areas on the right showing co-localization between ATG12 and VAMP2 in control cells and no co-localization in CALM knockdown cells. Quantification of ATG12-VAMP2 co-localization is shown on the graph as the Pearson’s coefficient (data are mean ±s.d.;*P<0.05; two-tailed *t*-test). Scale bars, 5 μm. (**b**) Western blot analysis of VAMP2, actin and LC3-II in HeLa cells where VAMP2 was knocked down, as indicated. The cells were starved in Hanks balanced salt solution (HBSS) and treated with Baf A1 as indicated. (SE, short exposure; LE, longer exposure.) Quantification of LC3-II/actin ratio is shown. Data are representative of three independent experiments and shown as mean ±s.d. (*P<0.05; NS, not significant, two-tailed *t*-test). (**c**) LC3 dot counting in CALM and VAMP2 knockdown cells. CALM or VAMP2 were knocked down in cells expressing GFP-LC3. The cells were kept in full medium or starved in HBSS for 4 h, fixed and subjected to automated fluorescence microscopy to score the number of LC3 dots per cell. The number of LC3 dots per cell (shown as mean ±s.d.) is shown on the graph for each condition (*n*≥300 cells per condition; BC, basal conditions). (*P<0.01; NS, not significant, two-tailed *t*-test). (**d**) Number of p62 dots per cell in VAMP2 knockdown. HeLa cells where VAMP2 was knocked down were fixed and subjected to microscopy after labelling endogenous p62 using specific antibody. The data represent the number of p62 dots per cell shown as mean ±s.d. (*P<0.05; two-tailed *t*-test; *n*≥300 cells per condition). (**e**) Percentage of Q74-expressing cells with aggregates in VAMP2 knockdown HeLa cells. Data are from one representative experiments performed in triplicate, out of three independent experiments and shown as mean ±s.d. (*P<0.05; two-tailed *t*-test). (**f**) Western blot analysis of VAMP2, actin and LC3-II in HeLa cells stably expressing VAMP2-HA wild type or VAMP-2-HA mutated in the CALM-binding site at different levels (wild-type clone 6, wt Cl6: low level; wild-type clone 11, wt Cl11: high level; mutant clone 11, AA Cl11: low level; mutant clone 12, AA Cl12: high level). The cells were treated with Baf A1 as indicated. (SE, short exposure; LE, longer exposure). Quantification of LC3-II/actin ratio is shown. Data are representative of three independent experiments and shown as mean ±s.d. (*P<0.05; two-tailed *t*-test).

Among the SNAREs whose endocytosis is regulated by CALM, no data are available on VAMP2 and autophagy. We confirmed that CALM regulates VAMP2 endocytosis by assessing its localization at the plasma membrane when CALM was knocked down in a stable cell line expressing VAMP2-HA (Fig. 5a)^26^,^27^. VAMP2 was localized to autophagic precursors (ATG12-positive) in both basal and autophagy-stimulated (starvation) conditions (Supplementary Fig. 4a), and CALM knockdown decreased the co-localization between ATG12 and VAMP2 (Fig. 5a), suggesting that VAMP2 may play a role in autophagic precursor formation/maturation. VAMP2 knockdown decreased LC3-II levels in cells in the presence of Baf A1, to a similar extent as we observed previously with SNAREs such as VAMP7 (ref. 16), suggesting that VAMP2 is involved in autophagosome formation (Fig. 5b). Distinct single siRNAs against VAMP2 also decreased LC3-II levels in Baf A1-treated cells (Supplementary Fig. 4b). The increased LC3-II levels in VAMP2-knockdown cells in the absence of Baf A1 may be due to decreased autophagosome degradation, in addition to decreased autophagosome formation, as seen with CALM depletion. We observed that the number of LC3 dots per cell increased in VAMP2 knockdown cells, in BCs (using two different siRNAs; Fig. 5c), suggesting a defect in autophagosome clearance. On starvation, the number of LC3 dots per cell increased in control cells, but we observed no further increase in VAMP2 knockdown cells (Fig. 5c). Thus, it appears that much similar to CALM, VAMP2 affects both autophagosome biosynthesis and autophagosome clearance. VAMP2 knockdown also increased the number of p62 dots per cell and the number of Q74 aggregates per cell (Fig. 5d,e), confirming a functional defect in autophagy substrate clearance.

Higher LC3-II levels were seen in a stable cell line overexpressing higher levels of VAMP2-HA, compared with one expressing lower levels of this protein (Supplementary Fig. 4c). As CALM affects VAMP2 endocytosis, we hypothesized that VAMP2 endocytosis mediated by CALM is required for effective autophagy. We used two pairs of stable cell lines with either high or low level expression of wild-type VAMP2-HA or a VAMP2-HA construct in which the CALM binding site was mutated (V43A and M46A, referred to as AA), trapping VAMP2 at the plasma membrane^27^. The cells expressing the VAMP2-HA mutant had significantly decreased autophagosome formation as demonstrated by the reduced LC3-II levels in the presence or absence of Baf A1 (Fig. 5f). The cells expressing the VAMP2-HA mutant spread more efficiently than wild-type cells or HeLa cells but did not have integrin β1-trafficking defects, which could have explained this phenotype (Supplementary Fig. 4d,e). Thus, the increased spreading of mutant cells is unlikely to be due to integrin trafficking but could be explained by decreased autophagic degradation of different proteins involved in cell migration, as observed previously^36^.

**Figure 6:**
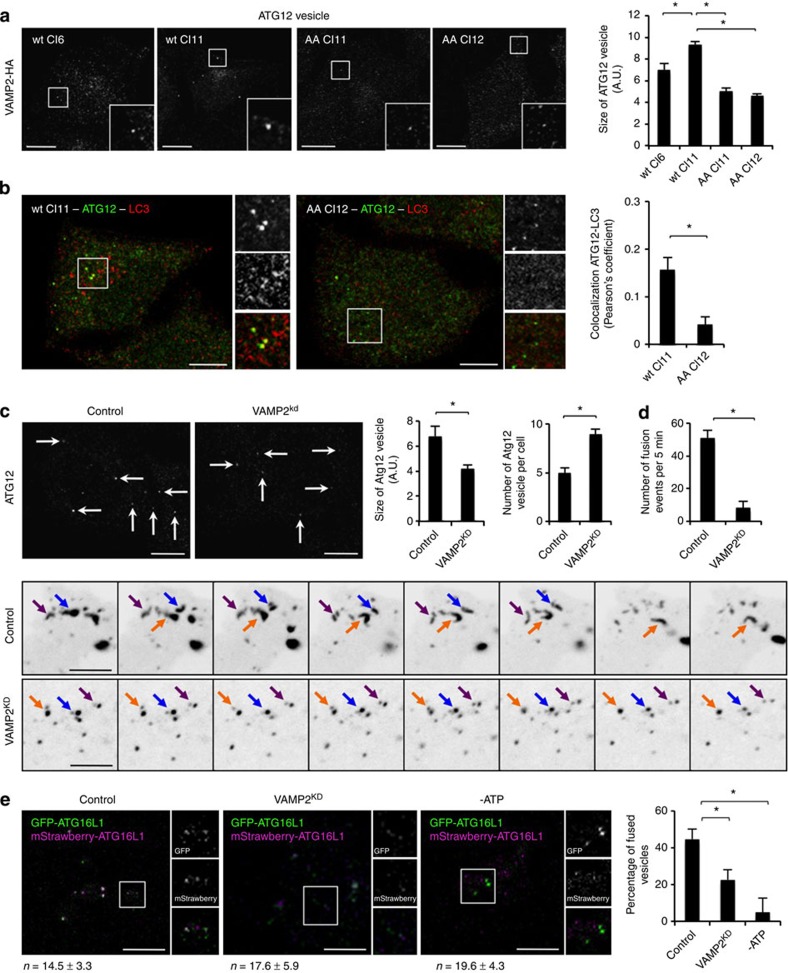
VAMP2 regulates autophagic precursor maturation. (**a**) Size of endogenous ATG12 vesicles in HeLa cells stably expressing either wild-type or mutant VAMP2. Confocal pictures are presented with magnified areas showing ATG12 vesicles. Data are representative of three independent experiments and shown as mean ±s.d. (*n*≥100 vesicles; *P<0.05; two tail *t*-test). Scale bars, 5 μm. (**b**) Co-localization between ATG12 and LC3 in HeLa cells stably expressing either wild-type clone 11 or mutant clone 12 VAMP2. Confocal pictures are presented with magnified areas showing ATG12-LC3 co-localization. The Pearson’s coefficient between ATG12 and LC3 is shown. Data are representative of three independent experiments and shown as mean ±s.d. (*n*≥100 vesicles; *P<0.05; two-tailed *t*-test). Scale bars, 5 μm. (**c**) Size and number of endogenous ATG12 vesicles in VAMP2-knockdown HeLa cells in starvation condition. Confocal pictures are shown with arrows indicating ATG12 vesicles. Data are representative of three independent experiments and shown as mean ±s.d. for the size of ATG12 vesicle. (*n*≥100 vesicles; *P<0.05; based two-tailed *t*-test). Scale bars, 5 μm. (**d**) Live-cell imaging of ATG16L1-GFP in VAMP2-knockdown HeLa cells. Confocal pictures from various time points of a 5-min movie are shown in inverted greyscale. Arrows indicate ATG16L1 vesicles. The number of fusion events per 5 min is shown. Data are representative of five movies and shown as mean ±s.d. (*P<0.05; two-tailed *t*-test). Scale bars, 5 μm. (**e**) *In vitro* fusion assay of post-nuclear supernatants from control and VAMP2 knockdown HeLa cells expressing either GFP-ATG16L1 or mStrawberry-ATG16L1. Confocal pictures are shown where ATG16L1-mStrawberry signal is shown in purple to enable better visualization. Fused vesicles appear in white. The ATP-negative condition, which prevents SNARE-dependent fusion, is also shown as a control of reaction. Magnified areas are shown to allow visualization of the vesicles. The percentage of fused vesicles (white) is represented. Data are representative of two independent experiments and shown as mean ±s.d. (*n*≥100 vesicles). Scale bars, 5 μm. (*P<0.05; two-tailed *t*-test). *n*=numbers of vesicles scored per field.

The cells with mutant VAMP2-HA also displayed smaller ATG12 vesicles compared with wild-type VAMP2-HA cells (Fig. 6a). Thus, we considered that VAMP2 may regulate homotypic fusion of ATG5/ATG12/ATG16L1 vesicles, as we observed with CALM. This possibility was reinforced when we also observed less co-localization between ATG12 and LC3 in VAMP2-HA mutant cells, compared with wild-type cells (Fig. 6b). This is a phenotype we observed previously when ATG16L1-positive autophagic precursor homotypic fusion was disrupted, and it correlates with impaired maturation of phagophore precursors into phagophores^16^. Indeed, VAMP2 knockdown reduced the size of ATG12 vesicles while increasing their number (Fig. 6c), similar to what we previously observed when we affected homotypic fusion of these vesicles^16^. We observed fewer homotypic fusion events of these vesicles assessed with live imaging (Fig. 6d and Supplementary Movies 3 and 4). Furthermore, VAMP2 knockdown also decreased the homotypic fusion efficiency of these vesicles *in vitro* (Fig. 6e), suggesting that VAMP2 is an additional SNARE that assists this process (consistent with its CALM-dependent localization on these vesicles). However, VAMP2 knockdown had no effect on ATG16L1-ATG9A co-localization, suggesting that it has no effect on heterotypic fusion (Supplementary Fig. 2e), a finding that mirrors our previous work showing that VAMP3 has no effect on ATG16L1 homotypic fusion^18^.

### CALM modulates autophagy and tau accumulation in *Drosophila*

**Figure 7:**
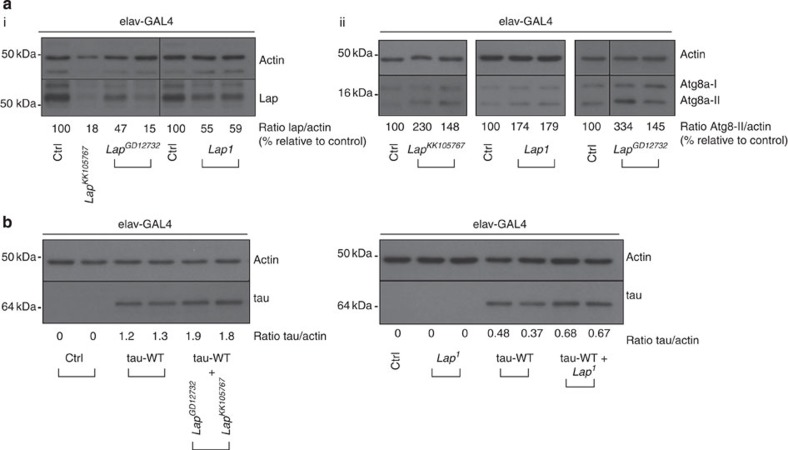
CALM regulates autophagy and tau degradation in *Drosophila*. (**a**) Western blotting showing downregulation of lap (i) or increase in Atg8a-II (ii) expression level in adult fly heads on *lap* downregulation using the *UAS-RNAi* lines *lap*^*GD12732*^ or *lap*^*KK105767*^, or the heterozygous allele *lap*^*1*^. Quantification of lap/actin and Atg8a-II/actin is shown. Genotypes: Control *w; elav-*GAL4/+; for the RNAi lines: *w; elav*-GAL4/ *lap*^*KK105767*^ and *w; elav-GAL4*/+; *lap*^*GD12732*^*/+*; for the *lap*^*1*^ allele: *w; elav-GAL4/+; lap*^*1*^*/+*. (**b**) Western blotting showing the accumulation of tau in *Drosophila* adult fly heads on lap downregulation using the *UAS-RNAi* lines *lap*^*GD12732*^ or *lap*^*KK105767*^, or the heterozygous allele *lap*^*1*^. Quantification of tau/actin is shown. Genotypes: Control *w; elav-*GAL4/+; for tau-WT: *w; elav*-GAL4/+; *UAS*-*tau-WT*/+; for RNAi lines: *w; elav*-GAL4/ *lap*^*KK105767*^*; UAS-tau-WT/+* and *w; elav-GAL4*/+; *lap*^*GD12732*^*/tau-WT*; for the *lap*^*1*^ allele: *w; elav-GAL4/+; lap*^*1*^*/+* or *w; elav-GAL4/+; lap*^*1*^*/UAS-tau-WT.*

GWAS studies have identified that CALM is associated with AD risk^20^,^21^. However, it is unclear how CALM impacts the development of AD. We hypothesized that it could be via its regulation of autophagy activities, which would impact tau clearance. *In vivo*, we observed that downregulation of the *Drosophila* CALM homologue, *lap*, using two independent RNA interference (RNAi) lines (*lap*^*GD12732*^ or *lap*^*KK105767*^, whose sequences do not overlap)^37^ or the heterozygous loss-of-function *lap*^*1*^ allele^38^ (Fig. 7ai), increases the level of Atg8a-II (Fig. 7aii), the closest *Drosophila* homologue of mammalian LC3-II (ref. 39), like mammalian CALM knockdown (Fig. 1). To study whether *lap* influences tau levels *in vivo*, we generated *Drosophila* overexpressing wild-type human tau (4R isoform). This transgenic line has no phenotype with regard to lethality, eye degeneration, locomotor activity, wing development and pseudopupil degeneration when we drive the transgene in diverse tissues (summarized in Supplementary Table 1 and Supplementary Fig. 5). This model expresses tau at much lower levels (<15%) compared with a model with overt phenotypes (Supplementary Fig. 5a)^40^. When we reduced *Drosophila lap* levels (using either RNAi or heterozygous allele *lap*^*1*^) in flies expressing human tau in neurons, we observed an increased human tau/actin ratio (Fig. 7b).

### CALM and autophagy contribute to tau clearance in zebrafish

**Figure 8:**
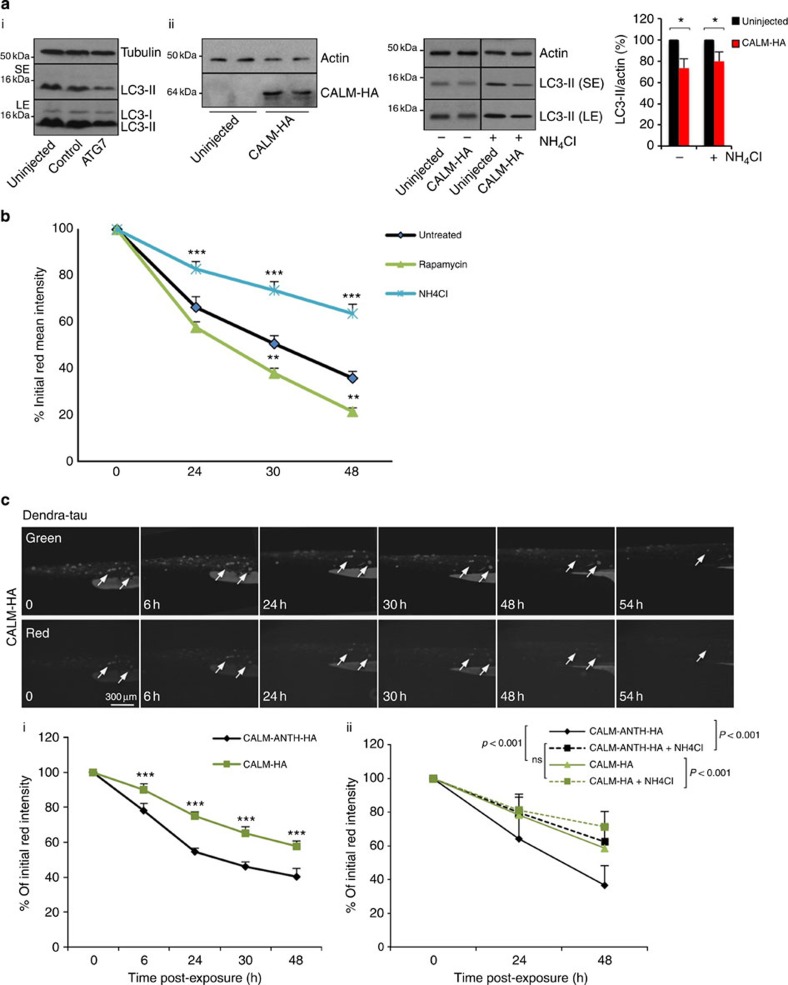
Autophagy and CALM contribute to tau degradation *in vivo*. (**a**i) Western blot analysis of tubulin, LC3-I and LC3-II in zebrafish larvae where ATG7 was downregulated, as indicated. (ii) Western blot analysis of CALM-HA, actin and LC3-II in zebrafish larvae where CALM-HA was expressed, as indicated. The larvae were treated with ammonium chloride (NH_4_Cl), as indicated. (SE, short exposure; LE, longer exposure). Quantification of LC3-II/actin ratio is shown. Data are representative of three independent experiments and shown as mean ±s.d. (*P<0.05; two-tailed *t*-test). (**b**) Modulation of autophagy alters Dendra-tau clearance dynamics. The fluorescence intensity of each individual cell was quantified at each timepoint (*n*≥ 31 cells, ≥9 larvae per treatment group) and mean cell intensity values for each drug treatment at each timepoint were calculated. Images were taken immediately after photoconversion and at 24, 30 and 48 h intervals thereafter. Rapamycin treatment significantly increased the rate of Dendra-tau clearance. Ammonium chloride (NH_4_Cl) treatment significantly decreases the rate of Dendra-tau clearance (**P<0.01, ***P<0.001, one-way analysis of variance (ANOVA)). Error bars are ±s.e.m. (**c**) Dendra-tau clearance in the presence of CALM: representative images of larvae with mosaic expression of Dendra-tau and full-length CALM taken immediately after photoconversion and at 6, 24, 30, 48 and 54 h after conversion. The fluorescence intensity of individual cells was quantified and mean fluorescent intensity of cells co-expressing Dendra-tau and either full-length CALM or Δ–ANTH CALM (CALM-ANTH-HA) constructs (*n*≥ 100 cells, ≥9 larvae per treatment group) at each time point was calculated. (i) Expression of full-length CALM significantly delayed the clearance of Dendra-tau at all time points compared with Δ–ANTH CALM (***P<0.001, one-way ANOVA). One graph, representative of three independent experiments, is presented. (CALM-ANTH-HA: 132 cells, 20 fishes; CALM-HA: 18 cells, 8 fishes). Another two experiments are shown in Supplementary Fig. S5C. Error bars are mean ±s.d. (ii) Treatment with ammonium chloride alters the dynamics of Dendra-tau clearance. Expression of full-length CALM significantly delayed the clearance of Dendra-tau at all time points compared with Δ–ANTH CALM, as observed in i (*P*<0.001, one-way ANOVA). However, treatment of Δ–ANTH CALM injected larvae with ammonium chloride slows the Dendra-tau clearance by 70% to a level comparable to that observed in larvae injected with full-length CALM. Ammonium chloride treatment of larvae injected with full-length CALM results in a modest (22%) decrease in Dendra-tau clearance, suggesting that CALM overexpression and ammonium chloride treatment have a cumulative effect (*n*≥25 cells, *n*≥9 larvae per treatment group). Error bars are mean ±s.d. Note that i and ii are distinct experiments.

Although the *Drosophila* tools enable study of the effects of CALM knockdown in relation to tau, we used zebrafish to examine the effects of CALM overexpression. We used this approach in favour of performing knockdown with morpholino oligonucleotides, as the two PICALM-like genes in zebrafish would probably necessitate silencing both genes, and the ‘gold standard’ validation for these assays (performing rescue experiments by co-injection of messenger RNA) would be practically impossible due to the effects resulting from overexpression. CALM overexpression decreased LC3-II levels in the zebrafish with or without ammonium chloride (NH_4_Cl), which mimics Baf A1 treatment (Fig. 8a), similar to what we observed in HeLa cells (Supplementary Fig. 1c).

To analyse whether CALM overexpression influenced the clearance of an AD-relevant autophagy substrate, we studied tau^5^. We generated an expression construct comprising a green-to-red photoconvertible fluorescent protein fused to human tau (Dendra-tau), which could be mosaically expressed in epithelial and muscle cells of zebrafish to measure tau clearance *in vivo*. Mosaic Dendra-tau fish were imaged before and after photoconversion and then daily, to assess clearance of the red (photoconverted) fluorescently tagged tau protein (Supplementary Fig. 6a,b). The autophagy-enhancing drugs rapamycin, clonidine and rilmenidine led to decreased abundance of red Dendra-tau (Fig. 8b and Supplementary Fig. 6b), and ammonium chloride (NH_4_Cl) treatment slowed the clearance of red Dendra-tau (Fig. 8b), suggesting that tau clearance is, at least in part, mediated via autophagy. Note that the NH_4_Cl in this assay is likely to be non-saturating, as complete autophagy/lysosome function compromise for a prolonged period in the whole organism would result in lethality. Co-injection of full-length CALM led to a marked reduction in the clearance of Dendra-tau compared with clearance with co-injection of CALM expressing the ANTH domain (Fig. 8c and Supplementary Fig. 6c). To determine whether this delay in tau clearance was a result of defective autophagy, zebrafish larvae injected with CALM and Dendra-tau were treated with ammonium chloride. Ammonium chloride treatment caused a 70% increase in the levels of Dendra-tau in larvae co-injected with CALM expressing the ANTH domain, demonstrating that inhibition of autophagy alters the tau clearance dynamics to a level similar to that of larvae injected with full-length CALM (Fig. 8c). In addition, ammonium chloride also caused a modest (22%) decrease in the clearance of full-length CALM, suggesting that ammonium chloride or CALM overexpression alone reduce, but do not completely block, autophagy and that together they have an additive effect.

**Figure 9:**
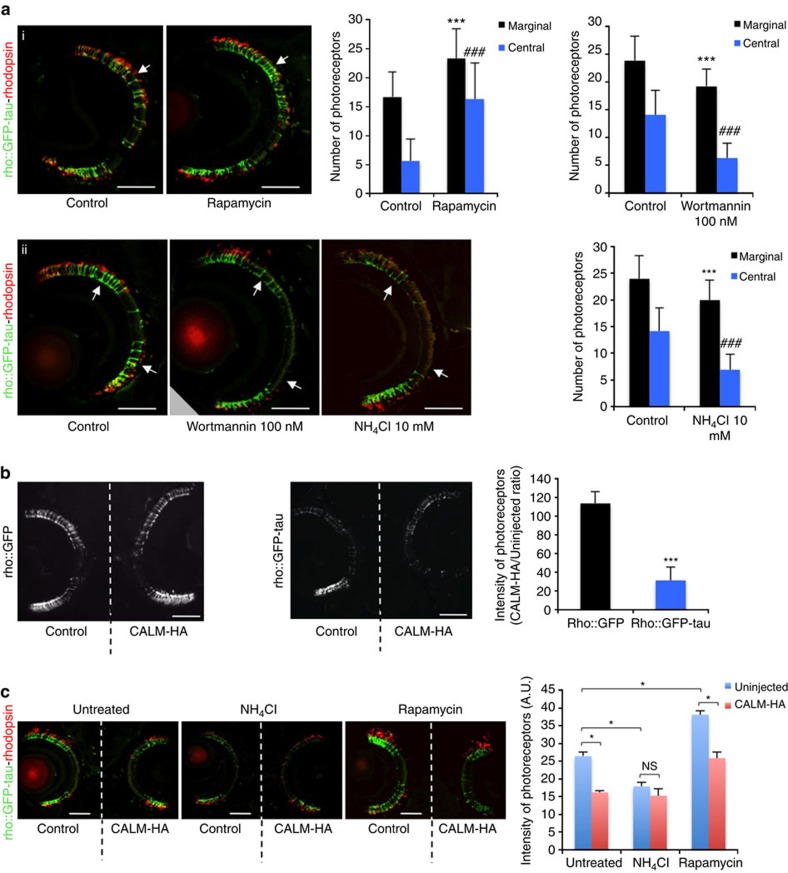
Autophagy and CALM modulate photoreceptor degeneration in zebrafish. (**a**) rho::GFP-tau fishes were incubated from 3 to 9 d.p.f. in either dimethyl sulphoxide (DMSO) or rapamycin (i) or from 3 to 7 d.p.f in EM alone or 10 mM ammonium chloride or 100 nM Wortmannin (ii). Images through the central retina at 9 d.p.f. (top panel; (i)) reveal rod degeneration (arrow) throughout the retina in control (DMSO-treated) larvae, whereas rod photoreceptors are present throughout the retina, particularly in the central region following treatment with rapamycin (arrow). Images taken through the central retina at 7 d.p.f. (bottom panel; (ii)) show normal photoreceptors in the marginal zones (arrows) and only limited numbers in the central region. NH_4_Cl exacerbates degeneration—photoreceptors are absent from the central retina and reduced/absent from marginal zones (arrows). Sections were stained with anti-rhodopsin (1D1) antibody. GFP labels whole rod photoreceptors, whereas rhodopsin is present in the rod outer segment. GFP co-localizes with the red rhodopsin label in all experimental conditions. Scale bars, 50 μm. Quantification of rod photoreceptor degeneration (*n*=10 larvae per group; ***P<0.001; ###*P*<0.001, two-tailed unpaired *t-*test). Error bars are mean ±s.d. (**b**) Full-length CALM electroporation into rho::GFP larvae did not cause degeneration., while full-length CALM electroporation into rho::GFP-tau larvae exacerbated photoreceptor degeneration. Central retina sections in the region of the optic nerve head are presented. Scale bars, 50 μm. Data represent the ratio (in %) of the intensity of the GFP signal between CALM-HA electroporated eye versus control eye for five individuals per transgenic line. ***P<0.05; two-tailed *t*-test. Error bars are mean ±s.d. (**c**) NH_4_Cl treatment of rho::GFP-tau immediately after unilateral CALM electroporation caused photoreceptor degenerationon the control (non-electroporated) side but did not alter the degeneration caused by CALM electroporation. Rapamycin treatment of rho::GFP-tau immediately after unilateral CALM electroporation rescued photoreceptors on the control (non-electroporated) side but not on the CALM-electroporated side. To demonstrate that loss of GFP corresponds to loss of photoreceptors, sections were stained with anti-rhodopsin (1D1) antibody. Wilcoxon signed rank test was used to compare the left eye versus the right eye of the same fish; Mann–Whitney test was used to compare drug treatment in the right eye of different fishes. *P<0.05. Scale bars, 50 μm. Error bars are mean ±s.e.m.

**Figure 10:**
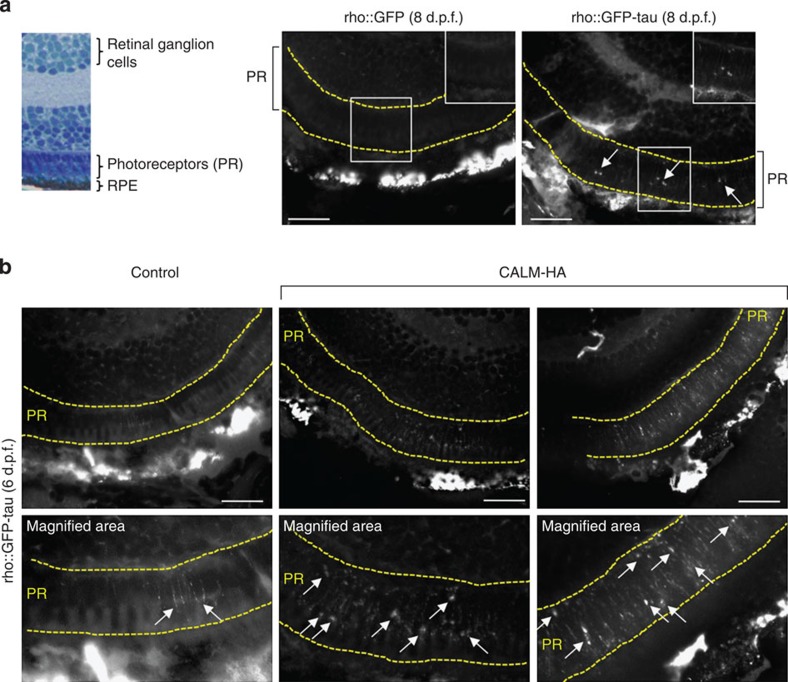
CALM modulates the formation of tau-positive tangles in zebrafish. (**a**) Histological section to demonstrate the individual cell layers of the zebrafish retina. The photoreceptor layer (PR, comprising rod and cone photoreceptors) lies immediately adjacent to the retinal pigment epithelium (RPE) at the outermost surface of the eye. Thioflavin-S labelling of retinal sections was used to identify neurofibrillary tangles in the photoreceptor layer (marked with yellow dotted lines). No labelling was observed in the retina of rho::GFP at 8 d.p.f., whereas distinct thioflavin-S-positive tangles (arrows) were observed in the photoreceptor layer of rho::GFP-tau fish. Note, the RPE is highly autofluorescent due to the presence of silver pigment. High power regions are shown in the top right of each panel. (**b**) Unilateral electroporation of CALM into the retina of rho::GFP-tau zebrafish resulted in a marked increase in thioflavin-S positive tangles in the electroporated retina in the photoreceptor layer (PR) compared with the control side. Top panel are lower magnification images to show the retinal cell layers. Thioflavin-S labelling is restricted to the PR layer. Note the RPE is highly autofluorescent due to the presence of silver pigment. Bottom panel are higher magnification images to show individual thioflavin-S tangles the largest of which are indicated by arrows.

To examine the consequences of CALM overexpression on tau toxicity, we developed a zebrafish model expressing enhanced GFP (EGFP)-tagged human tau under the control of the rhodopsin promoter to drive expression in the rod photoreceptors (rho::GFP-tau). We observed normal development of the rod photoreceptors expressing EGFP-tau from 3 days post fertilization (d.p.f.) to 6 d.p.f., then degeneration from 7 d.p.f. onwards, initially within the central retinal region and then at the margins (Supplementary Fig. 7a,b). Western blotting and immunohistochemistry for rhodopsin, the major component of the rod outer segment (Supplementary Fig. 7c), confirmed that the loss of EGFP-tau photoreceptors was truly degeneration of the rods rather than transgene downregulation. Accordingly, in subsequent experiments, we used the presence of EGFP-tau-positive photoreceptors as a proxy for assessing neurodegeneration. Before degeneration, we observed phosphorylation of the transgenic human tau protein at specific serine and threonine residues (Supplementary Fig. 7d); such phosphorylation is a hallmark of tau pathology in human and can be used as an indicator of disease progression^41^,^42^. The degeneration was confirmed by increased numbers of apoptotic cells in the retina of rho::GFP-tau compared with rho::GFP fish (a control transgenic line expressing GFP under the rhodopsin promoter) at 7 d.p.f. (Supplementary Fig. 7e). Rod photoreceptor degeneration was prevented by treatment with rapamycin (Fig. 9a) and exacerbated by the autophagy blockers wortmannin and NH_4_Cl (Fig. 9a), suggesting that autophagy manipulation can alter disease progression in this model, as described in other systems^10^. We developed a novel electroporation technique to deliver exogenous DNA to the photoreceptor layer of larval zebrafish (Supplementary Fig. 7f) and used this approach to overexpress CALM in the photoreceptors of rho::GFP-tau fish or rho::GFP fish to investigate the effects of CALM expression on tau-induced pathology. CALM overexpression in the photoreceptor layer accelerated rod photoreceptor degeneration and increased the number of apoptotic photoreceptors in rho::GFP-tau but did not cause any signs of pathology in the rho::GFP fish (Fig. 9b and Supplementary Figs 7e and 8a,b). To investigate whether neurofibrillary tangles were evident, we blocked cell death by treatment with the caspase inhibitor Z-VAD-FMK, to prolong photoreceptor survival. Thioflavin-S-positive tangles were observed in the photoreceptor layer of rho::GFP-tau fish, but not rho::GFP fish (Fig. 10). The accelerated degeneration following CALM overexpression was associated with increased tau phosphorylation and accumulation of thioflavin-*S*-positive tangles (Fig. 10b and Supplementary Fig. 8c). To validate that this degeneration was caused by autophagic impairment, we treated electroporated fish with autophagy-inducing (rapamycin) or -inhibiting (NH_4_Cl) drugs that modulated tau toxicity (Fig. 9a). NH_4_Cl exacerbated the photoreceptor degeneration in the control eye (as observed previously, Fig. 9a) such that the control eye and the electroporated eye showed equal levels of degeneration (Fig. 9c). Rapamycin rescued photoreceptor degeneration in the control eye but was unable to rescue the degeneration in the electroporated eye (Fig. 9c). Although rapamycin can induce autophagy and rescue degeneration in the control eye, our data suggest that CALM disrupts autophagosome biogenesis and therefore rapamycin treatment is unable to rescue degeneration in the CALM-electroporated eye, as autophagy upregulation via target of rapamycin inhibition cannot overcome the deficit in autophagosome formation caused by CALM. These data suggest that the effects of CALM on tau toxicity in this model are autophagy dependent.

## Discussion

One of the challenges of the post-genomic era is to understand how genes identified in association studies have an impact on disease. Our study suggests that at least one relevant way that CALM may influence AD development is by regulating autophagy. This is likely to have pleiotropic effects, and at least one of these may be via tau accumulation. Tau is an autophagy substrate^5^ and its accumulation in aggregates is a better indicator of functional impairment than the amount of extracellular β-amyloid in AD^1^. Although known *ATG* genes have not been associated with AD, this is not necessarily surprising. First, to have a chance of seeing a signal for such genes in GWAS, one needs to have allelic variants present above a certain frequency (depending on sample size) that have an impact on autophagy activity. If no such variants exist at this frequency, then no valid signal will emerge. Second, hemizygous loss of most of these genes has no effect on autophagy in mice, and homozygous losses cause early embryonic lethality. Thus, one might expect to find that autophagy-associated proteins, which have indirect links with ATG proteins, may be more likely to be associated with risk in diseases where autophagy plays a regulatory role. Nevertheless, extensive data in model systems and in human brains suggest modifying roles for autophagy in AD pathogenesis^5^.

CALM knockdown affects autophagosome formation at two early steps of the pathway. First, it affects ATG5/ATG12/ATG16L1 vesicle formation by regulating endocytosis. Although compromised endocytosis may have multiple effects on neuronal health, it has clear consequences for autophagy. Specifically, it influences the uptake of plasma membrane lipid into autophagosome precursor structures^12^, which we reported by knocking down key components of the endocytic apparatus, such as the clathrin heavy chain or a component of the clathrin adaptor AP2. A second distinct effect of CALM is to influence the maturation and enlargement of phagophore precursors and thereby autophagosome biogenesis by regulating VAMP2 and VAMP3 endocytosis. These SNAREs associate with pre-phagophore vesicles in a CALM-dependent manner and are required for optimal homotypic fusion of ATG16L1 (VAMP2) vesicles and heterotypic ATG16L1-ATG9A fusion (VAMP3) *in vivo* and *in vitro*^16^. Thus, CALM regulates both the initial generation of ATG16L1-associated autophagosome precursors, as well as their fusion events, which are required for efficient progression of these structures to the phagophore stage where they can acquire ATG8 family members^16^. Our data (Fig. 4a,b) suggest that CALM-dependent VAMP endocytosis is an important contributor to its autophagy effects. However, the relative importance of the different SNAREs is difficult to assess, and it is possible that CALM additionally has an impact on autophagy by regulating other intracellular trafficking events. CALM knockdown also affects autophagosome degradation and it is likely that this is mediated via its effects on VAMP8, as this SNARE is known to regulate autophagosome–lysosome fusion. The dual effects of CALM deficiency on autophagy result in a larger number of autophagosomes in BCs but a failure to upregulate autophagosome biogenesis in response to starvation. This situation may be very similar to that occurring in AD brains^4^,^5^.

CALM overexpression also affects autophagy, but this appears to be independent of VAMP2, VAMP3 or VAMP8 endocytosis, and is restricted to compromise formation of autophagosomes. We confirmed this scenario in zebrafish, where CALM overexpression leads to an accumulation of tau and enhanced toxicity. This is associated with slower tau clearance *in vivo*. Indeed, the Dendra-tau zebrafish allowed us to measure tau clearance *in vivo* in a pulse-chase manner, and thus avoid the caveats of measuring steady-state tau levels in such models, which may be altered by other processes, such as cell death. The autophagy-inhibitory effects of CALM overexpression are likely to enhance tau toxicity by increasing the load of this toxic protein. However, it is possible that compromised autophagy may additionally increase the severity of disease in the CALM-overexpressing zebrafish via additional mechanisms that are independent of the tau levels (for example, impaired susceptibility to apoptotic insults). Such possibilities are, however, very difficult to parse out in the *in vivo* setting.

In summary, we provide a mechanistic link between a validated AD risk factor and tau accumulation, which correlates well with AD pathological and functional deficits. The effect of CALM locus in AD risk allele(s) on CALM activity has still not been elucidated. However, a recent study has shown that CALM is cleaved in AD brains and the level of uncleaved CALM is also diminished in these patients^25^. Our cell-based and *in vivo* data suggest that both reduced and excessive CALM levels can have an impact on autophagy and tau levels/clearance, thereby modulating tau toxicity. Although an autophagy defect is probably not the only consequence of CALM dysregulation that contributes to neurodegeneration in AD, this mechanism provides a link between the genetic risk factor and tau accumulation, a major pathological hallmark of disease.

## Methods

### Cell culture

HeLa cells (from American Type Culture Collection (ATCC)) were cultured in DMEM medium D6546 (Molecular Probes) containing 10% fetal bovine serum, supplemented with 2 mM L-glutamine and 100 U ml^−1^ penicillin/streptomycin in 5% CO_2_ at 37 °C. HeLa cells stably expressing VAMP2-HA (wild-type clone 6 and clone 11; mutant clone 11 and clone 12 carrying mutations: Val43 and Met46 mutated to alanines), VAMP3-HA and VAMP8-HA were cultured in DMEM D6546 containing 10% fetal bovine serum supplemented with 2 mM L-glutamine, 100 U ml^−1^ penicillin/streptomycin and 500 μg ml^−1^ G418 (Sigma) in 5% CO_2_ at 37 °C as previously described^27^. HeLa cells expressing CALM constructs (wild-type and mutant) via a retrovirus (Phoenix System) were cultured in DMEM D6546 containing 10% fetal bovine serum supplemented with 2 mM L-glutamine, 100 U ml^−1^ penicillin/streptomycin, 0.17 mg ml^−1^ hygromycin B and 500 μg ml^−1^ G418 (Sigma) in 5% CO_2_ at 37 °C as previously described^32^. HeLa cells stably expressing GFP-LC3 were cultured in DMEM D6546 containing 10% fetal bovine serum supplemented with 2 mM L-glutamine, 100 U ml^−1^ penicillin/streptomycin and 500 μg ml^−1^ G418 (Sigma) in 5% CO_2_ at 37 °C.

HEK293 cells (from ATCC) were grown in DMEM containing 10% fetal bovine serum, supplemented with 100 U ml^−1^ penicillin/streptomycin and puromycin (5 μg ml^−1^) in 5% CO_2_ at 37 °C. CAD (Cath.-a-differentiated; a central nervous system catecholaminergic cell line established from a brain tumour in a transgenic mouse expressing neuron-specific proteins and synaptic vesicle proteins^43^) cells generously provided by Dr D. Chikaraishi, Department of Neurobiology, Duke University Medical Center, Durham, North Carolina, USA, and grown in DMEM-F12 media, supplemented with 100 U ml^−1^ penicillin/streptomycin in 5% CO_2_ at 37 °C. Murine embryonic fibroblasts were grown in DMEM supplemented with non-essential amino acids (Invitrogen) and 100 U ml^−1^ penicillin/streptomycin in 5% CO_2_ at 37 °C as described in ref. 44.

### Antibodies and reagents

Antibodies include: rabbit anti-actin (Sigma; ½,000), rabbit anti-ATG12 (Cell Signalling; 2010; 1/100), goat anti-CALM (Santa Cruz Biotechnology; sc-6433; 1/500), rabbit anti PICALM (Sigma; 1/1,000), rabbit anti-LC3 for western blotting (Novus Biologicals; NB100-2220; ¼,000), rabbit anti LC3 (Cell Signaling; 2775; 1/1,000), mouse monoclonal anti-LC3 for immunofluorescence (Nanotools; clone 5F10; 1/200), mouse anti-GFP (Clontech; 632569; 1/1,000), mouse anti-p62 (BD Transduction Lab; 610833; 1/1,000), rabbit anti-p62 (Novus Biologicals; NBP1-49954; 1/1,000), goat polyclonal DyLight 680 anti-rabbit IgG (Licor; 1/4,000) mouse anti-HA (Covance; clone 16B12; 1/1,000), mouse anti-VAMP2 (SySy, clone 11C3; 1/500), rabbit anti-VAMP8 (SySy; 104302; 1/1,000), mouse anti-GAPDH (Abcam; ab8245; ½,000), rabbit anti-ATG9A (Abcam; 2975-1; 1/200) and mouse anti-phosphorylated tau (PHF1, Thermo; MN1020; 1/200).

Reagents include: Baf A1 (Sigma), cholera toxin subunit B conjugated to Alexa555 (Invitrogen), transferrin conjugated to Alexa647 (Invitrogen) and EGF conjugated to Alexa555 (Invitrogen).

### Plasmids

pGFP-ATG16L1, pmStrawberry-ATG16L1, pCALM-HA, mCherry-LC3, LAMP1-GFP and DsRed-tau 4R have been described elsewhere^26^,^45^,^46^,^47^,^48^.

### Cell transfection

The cells were seeded at 1–2 × 10^5^ per well in six-well plates and transfection was performed using Lipofectamine or TransIT-2020 (for DNA) or Lipofectamine 2000 (for siRNA and double transfections with DNA and siRNA) (Invitrogen, Mirus), using the manufacturer’s protocol. Pre-designed siRNA were ordered from Thermo Scientific (Dharmacon Technologies) (siRNA IDs: human CALM—ON-TARGETplus SMARTpool, human CALM—oligo 5—5′-ACAGTTGGCAGACAGTTTA-3′; L-004004-00-0005; VAMP2—ON-TARGETplus SMARTpool and ON-TARGETplus Set of 4, L-012498-00-0005 and LU-012498-00-0002, respectively; VAMP3—ON-TARGETplus SMARTpool, L-011934-00-0005; and VAMP8—ON-TARGETplus SMARTpool, L-013503-00-0005). In all experiments in this paper, we used a scramble siRNA as the control from Thermo Scientific (Dharmacon Technologies; D-001810-10-05).

HEK293 and HeLa cells were stably transfected with shRNAs encoding pRSMX_PG vectors. CAD cells were infected with shRNAs encoding pRSMX_PG retroviral vectors. Selection of stably transfected/infected cells was performed with puromycin (Sigma). Details regarding the shRNAs used have been described previously^44^. CALM rescue of murine embryonic fibroblasts was performed using human CALM complementary DNA subcloned into the bicistronic MSCV-IRES-eGFP retroviral vector^44^.

### Modulation of autophagy *in vitro*

To inhibit LC3-II degradation, cells were treated with Baf A1 diluted in cell culture media to a working concentration of 400 nM for 4–6 h, which is saturating for this effect. To induce autophagy in a mammalian target of rapamycin-dependent manner, cells were amino acid- and serum-starved or only serum-starved in Hanks balanced salt solution (Sigma) for 1 to 4 h.

### Western blotting *in vitro*

Cells were collected, rinsed with PBS and lysed on ice for 30 min in PBS containing 1% Triton X-100 and complete protease inhibitor cocktail (Roche). Lysates were centrifuged at 15,000 r.p.m. for 5 min at 4 °C and supernatants were resolved by SDS–PAGE and transferred to polyvinylidene difluoride membranes. The membranes were blocked with TBST (TBS 0.1% Tween-20) containing 1% non-fat dry milk and were then incubated overnight at room temperature with primary antibody-diluted TBST. Membranes were washed with TBST, incubated for 1 h at room temperature with 2,500 × dilutions of horseradish peroxidase-conjugated secondary antibodies (GE Healthcare Bioscience) in TBST containing 1% non-fat dry milk and washed. Immunoreactive bands were then detected using ECL (GE Healthcare Bioscience) or by Odyssey infrared fluorescence imaging (LI-COR Biosciences). Cells were collected, spun down at 4,200 r.p.m. at 4 °C for 10 min. Cells were lysed in 2 × Laemmli buffer at a 1:1 dilution with PBS. Lysates were resolved by SDS–PAGE electrophoresis (12.5% gels) and transferred to polyvinylidene difluoride membranes. Membranes were incubated with the appropriate antibody overnight in Odyssey Blocking Buffer (LI-COR) with 0.1% Tween-20. Excess antibody was removed by performing three 10-min washes with TBST (0.1% Tween-20). Secondary antibody incubation was performed in Odyssey Blocking Buffer with 0.1% Tween-20 and 0.02% SDS for 30 min. The membranes were washed three times for 5 min each. Quantification of proteins normalized to actin or GAPDH (glyceraldehydes 3-phosphate dehydrogenase) was performed using an Odyssey Infrared Imaging System. Full blotting images are shown in Supplementary Fig. 9.

### Fluorescence and immunofluorescence microscopy *in vitro*

For immunofluorescence microscopy, cells were cultured on coverslips, fixed with 4% paraformaldehyde (PFA) in PBS for 5 min (for anti-p62, -CALM, -ATG12, -HA and -VAMP2 antibody) or with ice-cold methanol for 5 min (for anti-ATG12, -CALM and -LC3 antibodies), and permeabilized with 0.1% Triton X-100 in PBS for 5 min. Coverslips were incubated with primary antibodies for 2 h (room temperature) to 24 h (4 °C), washed three times with PBS and incubated with secondary antibodies for 30 min. Samples were mounted using ProLong Gold antifade reagent with or without DAPI (4′,6-diamidino-2-phenylindole; Invitrogen) and observed using a Zeiss LSM710 laser confocal microscope. Automatic counting of ATG12, LC3, Transferrin, EGF and p62 vesicles was performed using the Thermo Scientific Cellomics ArrayScan VTI HCS Reader and the Spot Detector Bioapplication protocole version 3.

### Live-cell imaging

For live-cell imaging, HeLa cells were seeded on 42-mm glass cover slips (PeCon, GmbH, Germany) at a density of ~1.5 × 10^5^ cells per cover slip. Cells were mounted in a POC chamber (PeCon GmbH) after which they were imaged immediately at 37 °C. Imaging was performed on a Zeiss Axiovert 200 M microscope with a LSM 710 confocal attachment using a × 63 1.4 numerical aperture Plan Apochromat oil-immersion lens. Laser lines at 488 nm (GFP-ATG16L1) were used. Laser power was kept at a minimum to minimize photobleaching and photocytotoxicity.

### *In vitro* fusion assay of ATG16L1 vesicles

The assay was performed using the protocol described previously^16^. Briefly, two post-nuclear supernatant from HeLa cells expressing either GFP-ATG16L1 or mStrawberry-ATG16L1 were mixed for 10–60 min in the presence of ATP and an ATP regenerative system, immobilized on glass coverslips and imaged by confocal microscopy, allowing visualization of double-labelled vesicles.

### Vesicle size assays

For measuring the size of ATG12 vesicles, two methods were used. The first method is based on the use of the Thermo Scientific Cellomics ArrayScan VTI HCS (High Content Analysis System) Reader and the Spot Detector Bioapplication protocol version 3, which allowed us to measure the size of more than 10,000 vesicles per sample. The resolution of the HCS is around 400 nm.

The second method is based on the use of a confocal microscope and image processing using ImageJ. The size of the vesicles were analysed using the Analyze Particles protocol. The resolution of this method is around of 150 nm. The data were statistically analysed using Mann–Whitney test.

### Time-course endocytosis assay

Cells were collected and resuspended in ice-cold serum-free CO_2_-independent medium and centrifuged for 1 min at 7,000 *g* and 4 °C, after which they were resuspended in 300 μl of SFM containing Alexa-647 transferrin (Molecular Probes) and incubated on ice for 5 min for prebinding. They were then incubated for different time (2, 5, 10 and 15 min) at 37 °C to allow internalization. The cells were chilled on ice, centrifuged and washed twice with 700 μl PBS, fixed with PFA (4% in PBS) and analysed by flow cytometry.

### Immuno-gold electron microscopy

HeLa cells stably expressing GFP-ATG16L1 for 20 h were then fixed with a mixture of 2% PFA and 0.2% glutaraldehyde in PBS for 2 h, at room temperature. Cells were then prepared for ultrathin cryosectioning and immunogold labelled, as previously described^49^. Briefly, fixed cells were washed once in PBS/0.02 M glycine, after which cells were scraped in 12% gelatin in PBS and embedded in the same solution. The cell-gelatin was cut into 1-mm blocks, infiltrated with 2.3 M sucrose at 4 °C, mounted on aluminum pins and frozen in liquid nitrogen. Ultra-thin cryosections were picked up in a mixture of 50% sucrose and 50% methyl cellulose, and incubated with anti-CALM and anti-GFP, and revealed with 15 nm and 10 nm protein A gold (Utrecht).

### Cell spreading assay

Cell spreading assay was performed as previously described using an automated fluorescence microscope^50^.

### Integrin-trafficking assay

The level of integrin β1 at the cell surface was measured by flow cytometry after immunostaining with a specific antibody against integrin β1 conjugated to fluorescein isothiocyanate (Abcam; ab21845) of cells fixed with PFA 4% for 2 min.

### Statistical analysis

Significance levels for comparisons between groups were determined with *t*-tests, repeated-measure, factorial analysis of variance or Mann–Whitney using the STATVIEW software, version 4.53 (Abacus Concepts, Berkeley, CA). For analyses of data where the controls’ absolute values vary in independent experiments (for example, LC3-II/actin), we have normalized control values in each experiment to 1 and used a paired *t-*test—this is the same as a one sample *t*-test.

### Maintenance of zebrafish stocks and collection of embryos

All zebrafish experiments were performed in accordance with Home Office Guidelines and local ethical committee approval. Zebrafish were reared under standard conditions on a 14 h light:10 h dark cycle. Embryos were collected from natural spawnings, staged according to established criteria^51^ and reared in embryo medium (EM) (5 mM NaCl, 0.17 mM KCl, 0.33 mM CaCl_2_, 0.33 mM Mg_2_SO_4,_ 5 mM HEPES) at 28.5 °C in the dark. Wild-type zebrafish were in-crossed descendants of the TL strain. The rhodopsin::EGFP line (Tg(rho.2:EGFP)^cu3^)^52^ was maintained as homozygous line.

### Microinjection of CALM in larval zebrafish

Full-length CALM cDNA was injected into fertilized zebrafish embryos at the one-cell stage. At 14 and 24 h.p.f., embryos (*n*=30 per condition) were transferred to chilled tubes, homogenized in lysis buffer then processed for western blotting as described above. In a separate set of experiments, embryos were either exposed to 100 mM ammonium chloride or kept in EM for 4 h before collection for western blotting.

### Generation of rhodopsin::EGFP-MAPT transgenic zebrafish

The fusion construct of EGFP-MAPT (0N4R)^53^ (kind gift from Dr Brian Anderton) was subcloned downstream of the zebrafish rhodopsin promoter^54^ into a modified pEX100T vector (ATCC). Circular DNA was co-injected with IsceI meganuclease into one-cell stage embryos and larvae with mosaic expression of EGFP in the retina were reared to adulthood. F0 mosaic fish were outcrossed to TL fish to identify germ line transmitting founders. F1 offspring were reared from 1 d.p.f. onwards in PTU and screened for EGFP expression in the rod photoreceptors at 4 d.p.f. F1 larvae with EGFP expression were reared to adulthood then incrossed to generate a homozygous transgenic line. The transgenic line is assigned Tg(rho:EGFP-tau)^cu7^ on the ZFIN database and referred to as rho::GFP-tau hereafter.

### Analysis of rod photoreceptor degeneration

Larvae were collected daily from 3 to 9 d.p.f., anaesthetized by immersion in 0.2 mg ml^−1^ 3-amino benzoic acid ethylester (MS222) then lysed for western blotting or fixed using 4% PFA in PBS at 4 °C for histological and immunohistochemical analysis. For western blotting to measure endogenous rhodopsin levels, *n*=30 larvae were collected per time point and membranes were probed with a mouse monoclonal anti-rhodopsin antibody (Zpr3, ZIRC) and with Arrestin, Zpr1 (cone-specific marker) as a loading control. For histology and immunohistochemical analysis, larvae were washed briefly in PBS, allowed to equilibrate in 30% sucrose in PBS then embedded in OCT medium (Tissue-Tek) and frozen on dry ice for subsequent cryosectioning. Sections were cut at 10-μm thickness using a cryostat (Bright Instruments) and mounted in 80% glycerol in PBS or Vectashield Hardset mounting medium (Vector Laboratories). Sections of the central retina, either side of the optic nerve head were imaged using a Zeiss Axioplan2 microscope, QImaging Retiga 2000 R digital camera and Q Imaging software. Images of retinal sections were divided into arcs of the central and margin regions (~110° and 40°), respectively, and the number of photoreceptors in each area was counted by viewing with the naked eye. Mean values for number of photoreceptors in each region of the retina was calculated for each time point. IHC analysis using 1D1 (anti-zebrafish rhodopsin, a kind gift from Paul Linser, University of Florida, FL), AT8 (Pierce Biotechnology), AT270 (Pierce Biotechnology) or PHF1 (a kind gift from Dr Peter Davies, Albert Einstein College of Medicine of Yeshiva University, NY) primary antibodies and Alexa 568 or 594 (Invitrogen) secondary antibody was performed on cryosections (*n*=5 fish per antibody). TUNEL (terminal deoxynucleotidyl transferase dUTP nick end) labelling was performed using an *In Situ* Cell Death Detection kit, TMR red (Roche).

### Compound treatments in rho::GFP-tau zebrafish

Rho::GFP-tau larvae were reared from 3 to 9 d.p.f. in EM containing either 0.1% dimethyl sulphoxide or 30 μM rapamycin or from 3 to 7 d.p.f in EM alone, 10 mM ammonium chloride or 100 nM wortmannin. Compounds and EM were refreshed daily. At the end of the treatment period, larvae were anaesthetized and fixed with 4% PFA in PBS for the analysis of rod photoreceptor number.

### Electroporation in zebrafish

Electroporation was performed as previously described^55^ with the following modifications: to achieve delivery of DNA to the photoreceptor layer, the negative electrode was placed into the brain and positive electrode touching the surface of the lens. A 4:1 ratio of full-length CALM DNA:dsRed DNA was injected into the orbit (Fig. 3c) and electroporated into the photoreceptor layer of the right eye of anaesthetized 4 d.p.f. larvae. Larvae were dark-adapted before electroporation to ensure photoreceptors had intercalated into the pigment epithelium. Larvae were reared in EM for 24 h then viewed using an Olympus SZX12 stereofluorescent microscope to identify those with incorporation of dsRed in the photoreceptors of the right eye. Compound treatments (30 μM rapamycin or 10 mM ammonium chloride) were performed from 4 to 6 d.p.f. (as described above) with treatment commencing immediately after electroporation. At 6 d.p.f., larvae were anaesthetized, fixed and processed for cryosectioning as described above. To assess cell death and tau accumulation, electroporation was performed without dsRed DNA such that red fluorophores could be used in subsequent analysis. Sections of the central retina, either side of the optic nerve head were imaged using a Zeiss Axioplan2 microscope, QImaging Retiga 2000 R digital camera and Q Imaging software.

### Thioflavin-S staining in zebrafish

To inhibit death of the photoreceptors, larvae were treated from 4 d.p.f. onwards with the caspase inhibitor Z-VAD-FMK at 300 μM, a concentration previously shown to be effective at inhibiting cell death without causing toxicity or any adverse physiological effects^56^. Thioflavin-S staining was performed on cryosections using a modified Thioflavin-S staining protocol (described previously^57^).

### Generation and microinjection of Dendra-tau constructs

Wild-type human MAPT (2N4R) in pCDNA3.1 (a gift from Dr Li Gan) was subcloned into pDendra2 at *Kpn*I and *Apa*I sites. Dendra-tau was injected into zebrafish embryos at the one cell stage either alone or in 1:3 ratio with full-length CALM or Δ–ANTH CALM constructs. At 24 h.p.f., embryos were viewed using EGFP filter sets on an Olympus SZX12 stereofluorescent microscope to identify those with mosaic expression of Dendra-tau. Dendra fluorescent protein was photoconverted from green to red by exposure of whole larvae to 365 nm light at 48 h.p.f. Digital images were captured using GX Optical LED fluorescent microscope, GXCAM3.3 digital camera and GX Capture software before (M0) and after photoconversion (M1), and at defined time points thereafter to monitor Dendra-tau clearance.

### Compound treatment in Dendra-tau-injected zebrafish

Embryos injected with Dendra-tau were reared in EM containing either 0.1% dimethyl sulphoxide, 30 μM rapamycin, 50 μM rilmenidine, 3 μM clonidine or 1 mM ammonium chloride from 2 to 5–6 d.p.f. with drugs refreshed daily. Dendra fluorescent protein was photoconverted from green to red by exposure of whole larvae to 365 nm light at 48 h.p.f. Digital images were captured using before (M0) and after photoconversion (M1), and at 24 (M2), 48 (M3), 72 (M4) and 96 h (M5) time points thereafter to monitor Dendra-tau clearance.

### Quantification of Dendra-tau clearance in zebrafish

Digital images of fluorescent cells in larvae with Dendra-tau mosaic expression were analysed using ImageJ. The number of red fluorescent cells per larva was counted immediately after photoconversion and at each time point thereafter. A region of interest was generated around each cell with Dendra-tau expression and fluorescent intensity was measured using region of interest and integrated density functions. The fluorescent intensity of each cell was quantified at each time point and expressed as a percentage of the initial fluorescent intensity at M1 (immediately after photoconversion).

### Generation of tau-WT transgenic flies

The cDNA of wild-type human *MAPT (2N4R)* in *pCDNA3.1* (a gift from Dr Li Gan) was subcloned into the *pUASTattB*^58^ vector using the Gateway system (Invitrogen). The DNA was injected into embryos using the PhiC31 integrase system and the vector inserted on the second chromosome at the *ZH-51D* landing site^58^. The MAPT insertion was verified by sequencing across its 5′ and 3′ junctions with the *pUASTattB* vector, both before and after injection.

### Western blotting in *Drosophila*

To assess tau-WT expression level, 20 heads of flies expressing *elav-*GAL4 and tau-WT, crossed to either *lap UAS-RNAi* lines (*lap*^*GD12732*^ or *lap*^*KK105767*^, VDRC Stock Center, http://stockcenter.vdrc.at/control/main)^37^, *lap*^*1*^ (ref. 38) or wild-type control lines, were lysed in sample buffer (4% SDS, 20% glycerol and 0.125 M Tris HCl, pH ~6.8). Wild-type control lines were *w*^*1118*^ (VDRC stocks 60000 and 60100 for *lap*^*GD12732*^ and *lap*^*KK105767*^, respectively), or *Canton S* (gift of Simon Collier) for *lap*^*1*^. The tau-WT transgenic line (gift of Mel Feany) of Wittmann *et al*.^40^ was used as a positive control. Lysed supernatant was run on SDS–PAGE using standard techniques. Proteins were detected using rabbit anti-tau (1:1,000, ab74391, Abcam), rabbit anti-actin (1:5,000, A2066, Sigma), rabbit anti-Atg8a (1:5,000, a kind gift from Dr G. Juhász)^59^. IRDye 800CW anti-rabbit and IRDye 680LT anti-rabbit were used as secondary antibodies and the signal detected via LI-COR Odyssey Fc dual-mode imaging system. To assess lap expression levels, SDS–PAGE western blottings were probed with a guinea pig anti-lap polyclonal antiserum (a kind gift from Dr B. Zhang, similar to the rat anti-serum used previously^38^) used at a dilution of 1:3,000 with a secondary horseradish peroxidase-labelled anti-guinea pig IgG. Full blotting images are shown in Supplementary Fig. 9.

### Characterisation of *Drosophila* tau-WT transgenic lines

Virgins of the drivers tubulin-GAL4 (P{tubP-GAL4}LL7 (ref. 60)), GMR-GAL4 (P{GAL4-ninaE.GMR}12 (ref. 61)), eyeless-GAL4 (ey-GAL4, P{GAL4-ey.H}3-8 (ref. 62)), elav-GAL4 (elav-GAL4^C155^ (ref. 63)), D42-GAL4 (P{GawB}D42 (ref. 64)), CCAP-GAL4 (P{CCAP-GAL4.P}16 and P{CCAP-GAL4.P}9 (ref. 65)), NSyb-GAL4 1-2 (gift of Dr Julie Simpson) were crossed with males carrying UAS-tau-WT.

### *Drosophila lap* KK-RNAi line *lap*^*KK105767*^

We verified that the *lap*^*KK105767*^*UAS-RNAi* line was not inserted at the landing site that affected the *tio* gene^66^ by crossing virgins of *elav-GAL4*^*C155*^ (ref. 63) with either *lap*^*KK105767*^ or *w*^*1118*^ (60100 stock, VDRC Stock Center, http://stockcenter.vdrc.at/control/main) males. The progeny of both crosses had normally inflated wings, suggesting that they harbour the targeted insertion at the anticipated genomic site^66^.

## Author contributions

D.C.R. conceived and supervised the project. K.M., A.F., S.I., A.L.R., J.L.M., M.J.-S., C.F.B., C.P., E.Z., F.S., C.P.C. and M.B. acquired the data. D.C.R., K.M., A.F., S.I., C.J.O’.K. and D.S.W. wrote the article. C.J.O’.K. supervised *Drosophila* work and D.S.W. supervised some of the cell work. All authors interpreted the data. S.I., A.L.R. and J.L.M. contributed similarly to the work.

## Additional information

**How to cite this article:** Moreau, K. *et al.* PICALM modulates autophagy activity and tau accumulation. *Nat. Commun.* 5:4998 doi: 10.1038/ncomms5998 (2014).

## Supplementary Material

Supplementary Figures, Table and References Supplementary Figures 1-9, Supplementary Table 1 and Supplementary References.

Supplementary Movie 1 HeLa cells transfected with two rounds of control siRNA for 4 days were then transfected for 20 h with GFP-ATG16L1. Cells were subjected to live cell imaging. Time series: 30 frames/sec for 5 min.

Supplementary Movie 2 HeLa cells transfected with two rounds of CALM siRNA for 4 days were then transfected for 20 h with GFP-ATG16L1. Cells were subjected to live cell imaging. Time series: 30 frames/sec for 5 min.

Supplementary Movie 3 HeLa cells transfected with two rounds of control siRNA for 4 days were then transfected for 20 h with GFP-ATG16L1. Cells were subjected to live cell imaging. Time series: 30 frames/sec for 5 min.

Supplementary Movie 4 HeLa cells transfected with two rounds of VAMP2 siRNA for 4 days were then transfected for 20 h with GFP-ATG16L1. Cells were subjected to live cell imaging. Time series: 30 frames/sec for 5 min.
