# Supplementary material for: PICALM modulates autophagy activity and tau accumulation
Source: Nat Commun. 2014 Sep 22;5:4998. doi: 10.1038/ncomms5998 (PMC4199285; doi:10.1038/ncomms5998)
Supplement: Supplementary Figures, Table and References — Supplementary Figures 1-9, Supplementary Table 1 and Supplementary References. [file ncomms5998-s1.pdf]

Supplementary information

Supplementary Figure 1: CALM regulates autophagy

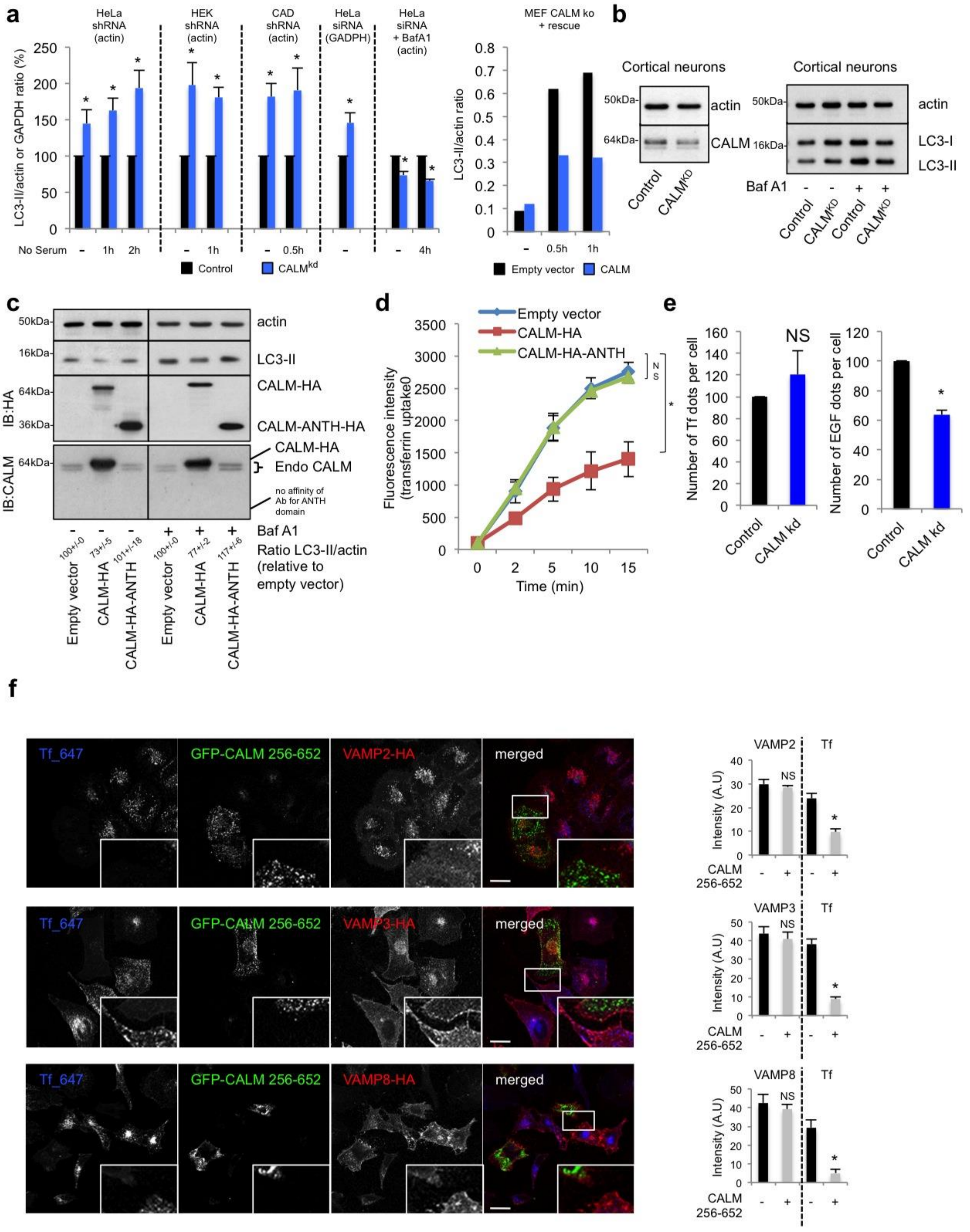

Supplementary Figure 1. CALM regulates autophagy.

(a). Quantification of LC3 levels in the experiment described in Figure 1A. Data are mean +/- SD (n > 3 experiments for each condition except of CALM knockout MEF where data shown is representative of results obtained in 3 independent clones of CALM knock out MEFs).

(b). Western blot analysis of CALM, actin and LC3-II in primary neurons where CALM was knocked down using siRNA, as indicated. The cells were treated with bafilomycin A1 (Baf A1) as indicated.

(c). Western blot analysis of actin, CALM and LC3-II in HeLa cells expressing an empty vector, CALM-HA wild type or CALM-HA without ANTH domain (CALM-HA-ANTH) for 20 h. The cells were treated with bafilomycin A1 (Baf A1) as indicated. The graph is representative of 4 independent experiments. Data are mean +/- SEM and are shown below the western blot.

(d). Transferrin uptake in HeLa cells expressing an empty vector, CALM-HA wild-type or CALM-HA without ANTH domain (CALM-HA-ANTH) for 20 h. Fluorescence intensity of transferrin inside the cells is shown. Data are representative of three independent experiments and shown as mean +/- S.D. *: p<0.05.

(e). Endocytosis of transferrin and EGF in CALM knockdown cells. HeLa cells where CALM was knocked down as indicated were loaded for 15 min with transferrin-Alexa647 and EGF-Alexa555, fixed and subjected to automatic microscopy. The graphs represent the number of transferrin and EGF dots per cell as mean +/- S.D. (n ≥ 200 cells per condition; *: $p<0.05$; NS: not significant, two-tailed *t*-test).

(f). SNARE and transferrin endocytosis in CALM overexpressing cells. HeLa cells stably expressing either VAMP2-HA, VAMP3-HA or VAMP8-HA were transfected with GFP-CALM 256-652 construct. Cells were incubated with transferrin conjugated with Alexa647 for 30 minutes before fixation. SNAREs were immunostained using specific antibodies without membrane permeabilization in order to detect plasma membrane pool. Confocal pictures are shown with magnified areas showing no effect of CALM expression on SNAREs endocytosis, while having a strong effect on transferrin internalization. Data represent intensity of SNAREs signal at plasma membrane or transferrin intensity in cells expressing GFP-CALM 256-652 as indicated as mean +/- SEM (n ≥ 200 cells per condition; *: $p<0.05$; NS: not significant, two-tailed *t*-test).

Supplementary Figure 2: CALM regulates autophagic precursors maturation

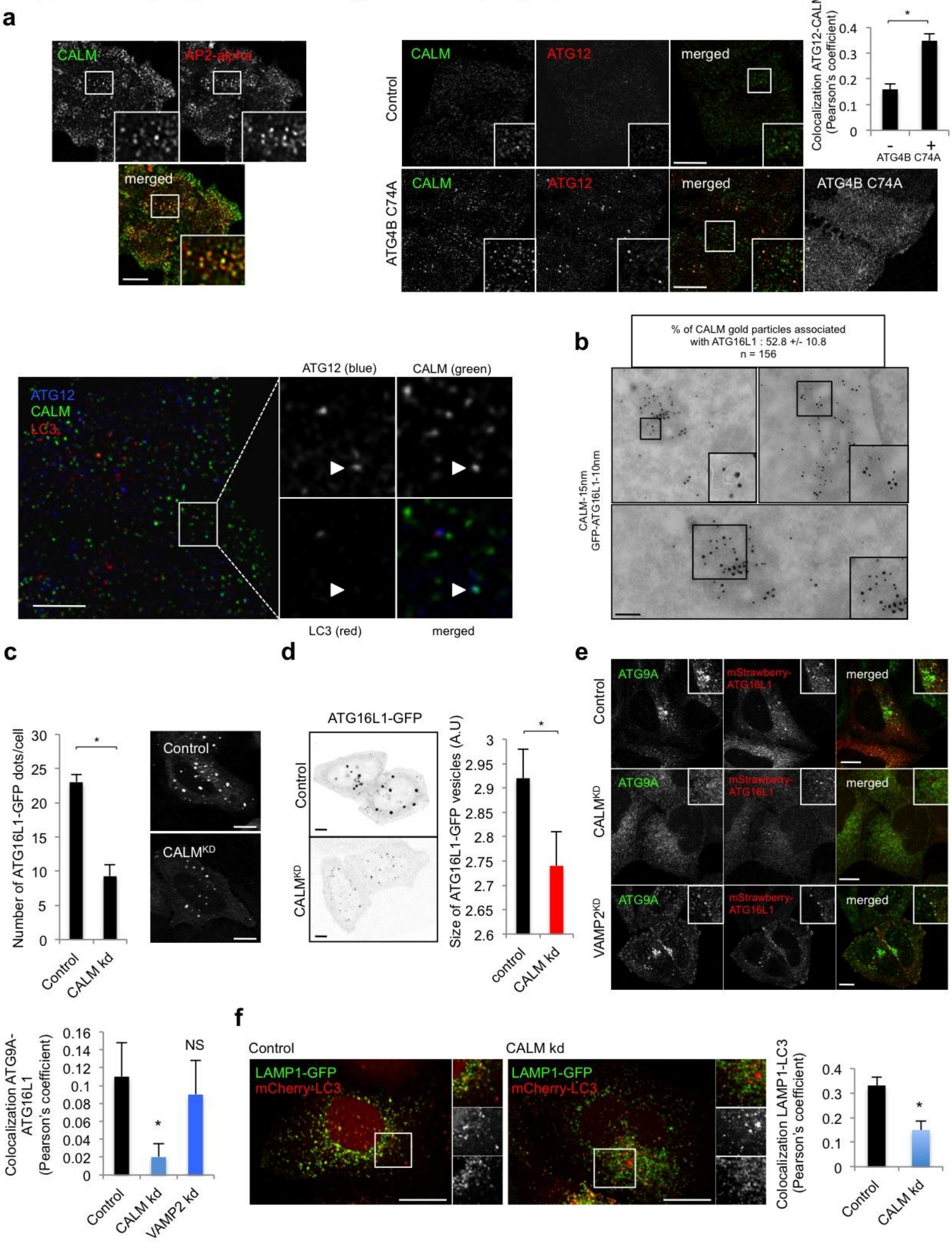

Supplementary Figure 2. CALM regulates autophagic precursor maturation.
(a). Colocalization between endogenous CALM and ATG12 in HeLa cells transfected with (bottom row) or without (top row) the ATG4B C74A-mStrawberry mutant. Confocal pictures are shown. The Pearson's coefficient between CALM and ATG12 is shown. Data are representative of three independent experiments and shown as mean +/- S.D. (n ≥ 20 cells; *: p<0.05; two-tailed t-test). Colocalization between endogenous CALM, ATG12 and LC3 are shown at the bottom where arrowheads indicate an ATG12-positive, CALM-positive and LC3-negative vesicle on the right panel. Scale bars, 5 µm.
(b). Immuno-electron microscopy of CALM (15 nm gold particles) and ATG16L1-GFP (10 nm gold particles) in HeLa cells. Magnified areas show a vesicle containing both CALM and ATG16L1. Scale bars, 100 nm.
(c). Formation of GFP-ATG16L1 vesicles in CALM knockdown HeLa cells. Confocal pictures are shown. Data shown is one representative experiment of three independent experiments. Data shown as mean +/- SEM (n ≥ 100 cells; *: p<0.01; two tail t-test). Scale bars, 5 µm.
(d). Size of GFP-ATG16L1 vesicles in CALM knockdown HeLa cells. Confocal pictures in gray inverted style are shown. Data shown are from one representative experiment of three independent experiments. Data shown as mean +/- SEM (n ≥ 100 cells; *: p<0.01; two-tailed t-test). AU: arbitrary unit. Scale bars, 5 µm.
(e). Colocalization between ATG9A and ATG16L1 in CALM and VAMP2 knockdown cells. HeLa cells knocked down for CALM or VAMP2 as indicated were transiently transfected with mStrawberry-ATG16L1, fixed and subjected to confocal microscopy. Confocal pictures are shown with magnified areas at the top of each picture. The graph represents the colocalization between ATG9A and ATG16L1 as a Pearson's coefficient. Data are mean +/- SD (n ≥ 50 cells; *: p<0.01; NS: not significant; two-tailed t-test). Scale bars, 5 µm.
(f). Colocalization between LAMP1 and LC3 in CALM knockdown cells. HeLa cells knocked down for CALM were transiently transfected with LAMP1-GFP and mCherry-LC3, fixed and subjected to confocal microscopy. Confocal pictures are shown with magnified areas on the right of each picture. The graph represents the colocalization between LAMP1 and LC3 as the Pearson's coefficient. Data are mean +/- SD (n ≥ 50 cells; *: p<0.01; two-tailed t-test). Scale bars, 5 µm.

Supplementary Figure 3: VAMP3 and VAMP8 modulate autophagy activity

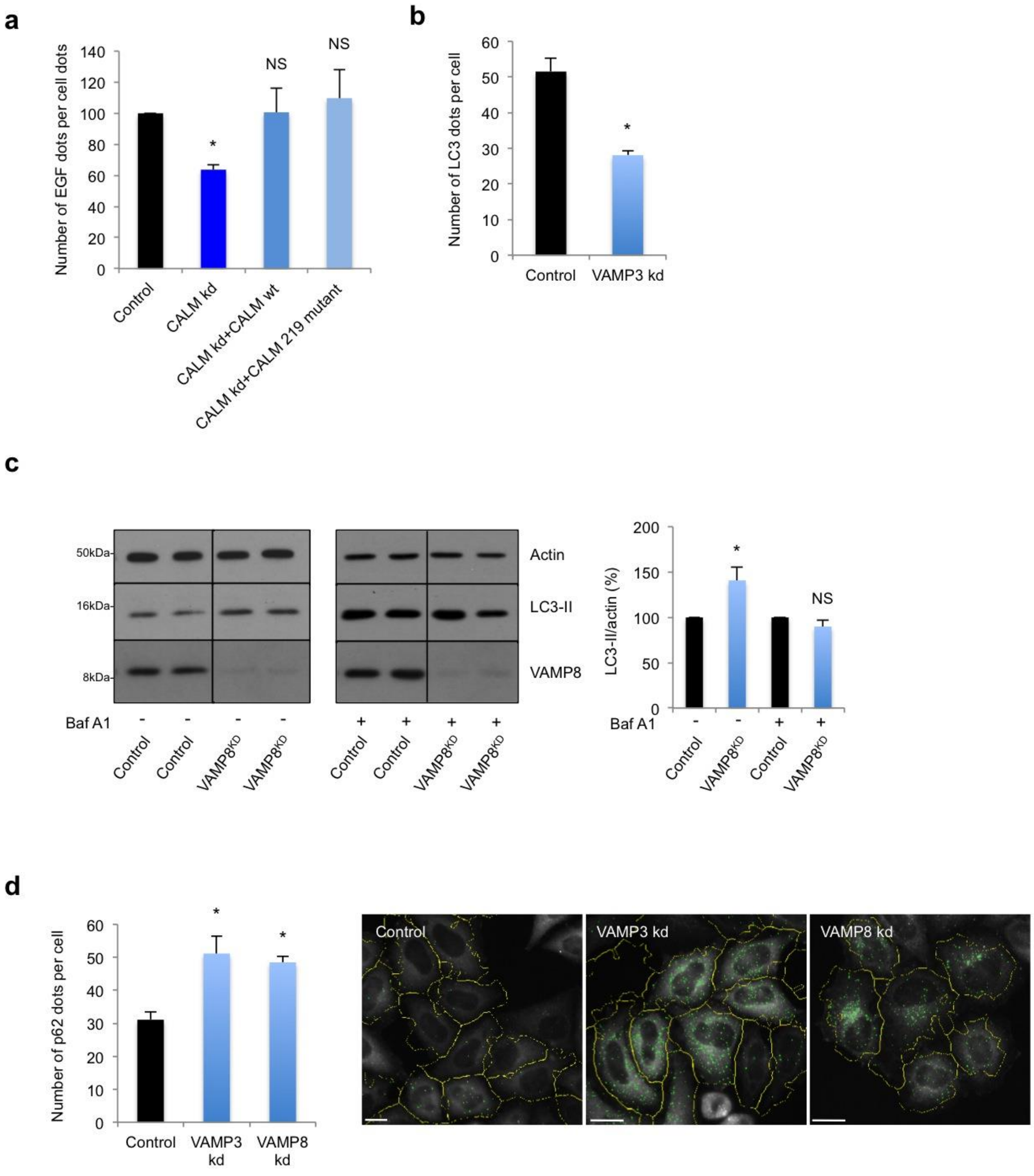

Supplementary Figure 3. VAMP3 and VAMP8 modulate autophagy activity.
(a). Endocytosis of EGF in siRNA resistant CALM. HeLa cells expressing or not a siRNA resistant form of CALM wild type (wt) or CALM mutant (219 mutant) where CALM was knocked down as indicated were loaded for 15 min with EGF-Alexa555, fixed and subjected to automatic microscopy. The graphs represent the number of EGF dots per cell relative to the control as mean +/- S.D. (n ≥ 200 cells per condition; *: p<0.05; NS: not significant, two-tailed *t*-test).
(b). Number of LC3 dots per cell in VAMP3 knockdown. HeLa cells where VAMP3 was knocked down were fixed and subjected to automated microscopy. The data

represent the number of LC3 dots per cell shown as mean +/- S.D. (*: $p<0.05$; two-tailed *t*-test; n ≥ 300 cells per condition).

(c). Western blot analysis of VAMP8, actin and LC3-II in HeLa cells where VAMP8 was knocked down. The cells were treated with Baf A1 as indicated. Quantification of LC3-II/actin ratio is shown. Data are representative of 4 independent experiments and shown as mean +/- S.D. (*: $p<0.05$; NS: not significant; two-tailed *t*-test).

(d). Number of p62 dots per cell in VAMP3 and VAMP8 knockdown. HeLa cells where VAMP3 or VAMP8 was knocked down were fixed and subjected to microscopy after labelling endogenous p62 using specific antibody. The data represent the number of p62 dots per cell shown as mean +/- S.D. (*: $p<0.05$; two-tailed *t*-test; n ≥ 300 cells per condition). Representative automated microscopy pictures are shown for each condition. Scale bars, 10 µm.

Supplementary Figure 4: VAMP2 regulates autophagy

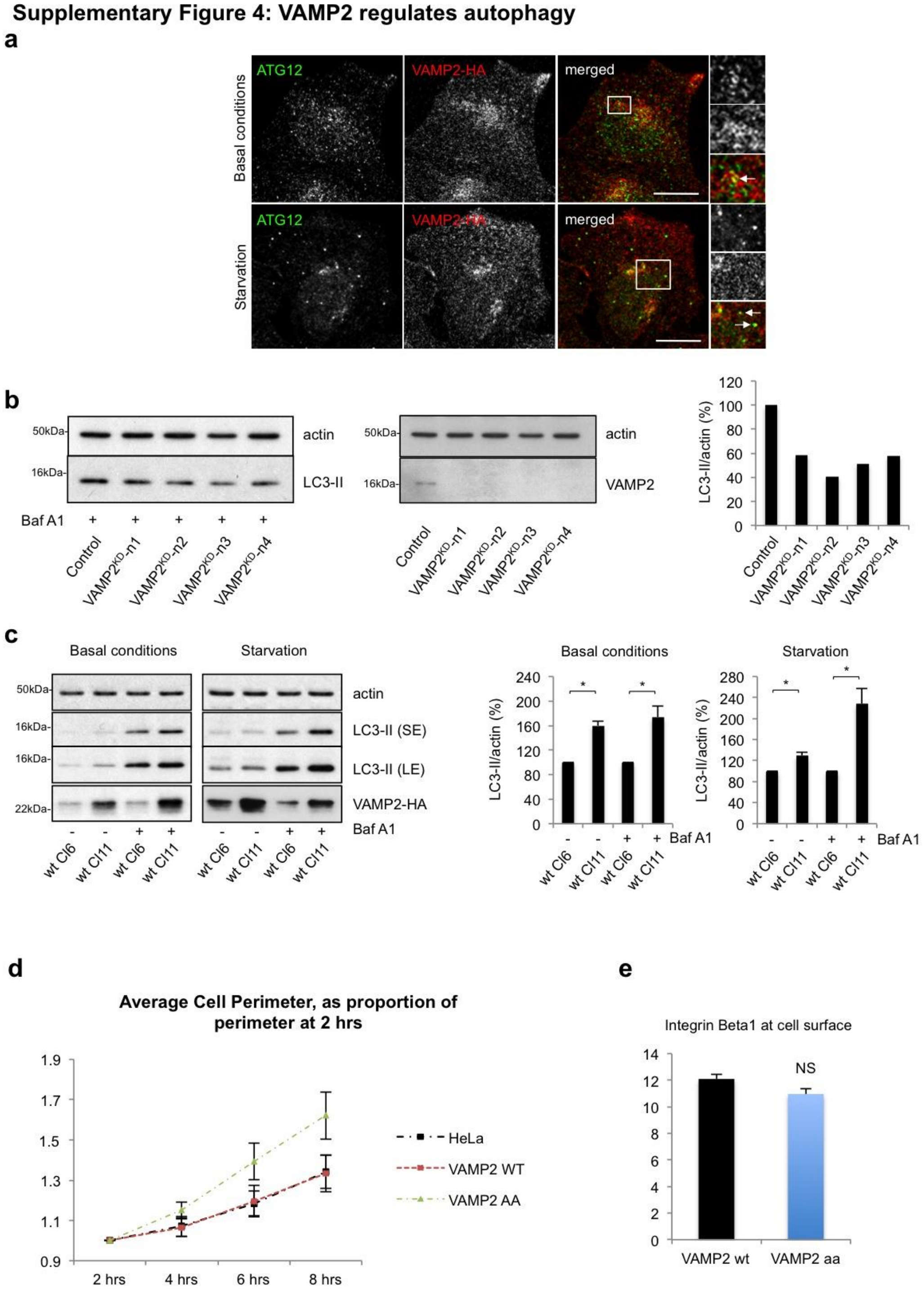

Supplementary Figure 4. VAMP2 regulates autophagy.

(a). Colocalization between ATG12 and VAMP2-HA in HeLa cells overexpressing VAMP2-HA cultured in basal conditions or in autophagy-stimulated conditions with starvation for 2 h. Confocal pictures are shown with magnified areas with arrows indicating ATG12/VAMP2 colocalization. Scale bars, 5 µm.

(b). Western blot analysis of VAMP2, actin and LC3-II in HeLa cells where VAMP2 was knocked down using distinct single siRNAs. The cells were starved in HBSS and treated with bafilomycin A1 (Baf A1) as indicated. Quantification of LC3-II/actin ratio is shown.

(c). Western blot analysis of VAMP2, actin and LC3-II in HeLa cells stably expressing VAMP2-HA at different levels (wild type clone 6, wt Cl6: low level; wild type clone 11, wt Cl11: high level). The cells were starved in HBSS and treated with bafilomycin A1 (Baf A1), as indicated. (SE: short exposure; LE: longer exposure). Quantification of LC3-II/actin ratio is shown. Data are representative of three independent experiments and shown as mean +/- S.D. (*: $p<0.05$; two-tailed *t*-test).
(d). Cell spreading in VAMP2 stably expressing cell. Cells expressing VAMP2 wild-type, VAMP2 mutant in CALM binding site or HeLa were fixed at different timepoints and analysed for cell spreading using automatic microscope. Data are average cell perimeter, as proportion of perimeter at 2h as mean +/-SEM (n=3 experiments).
(e). Integrin beta1 trafficking to plasma membrane. Cells expressing VAMP2 wild-type or VAMP2 mutant in CALM binding site were fixed, incubated for 2h with anti-integrin beta1 antibody conjugated to FITC at room temperature and analysed by flow cytometry. Data are mean +/- SEM of fluorescence intensity (cell surface integrin beta1) (n=3).

Supplementary Figure 5: tau-WT expression levels in Drosophila.

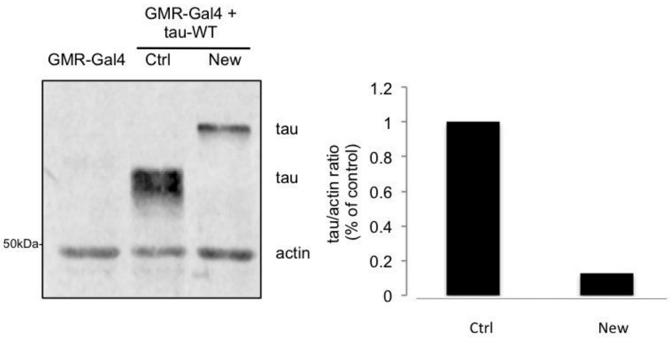

Supplementary Figure 5. tau-WT expression levels in *Drosophila*.
Western blot shows different tau-WT expression levels when expressed in the eye using GMR-GAL4 at 25°C (new). Flies expressing a tau-WT construct from Wittmann et al. [1] grown at 25°C were used as a positive control (Ctrl). Expression levels are quantified in the graph on the right. Note that flies from Wittmann et al. [1] express a shorter isoform (3R) than the new line we created (4R), explaining the different migration of tau on the western blot.

Supplementary Figure 6: CALM regulates tau clearance in zebrafish

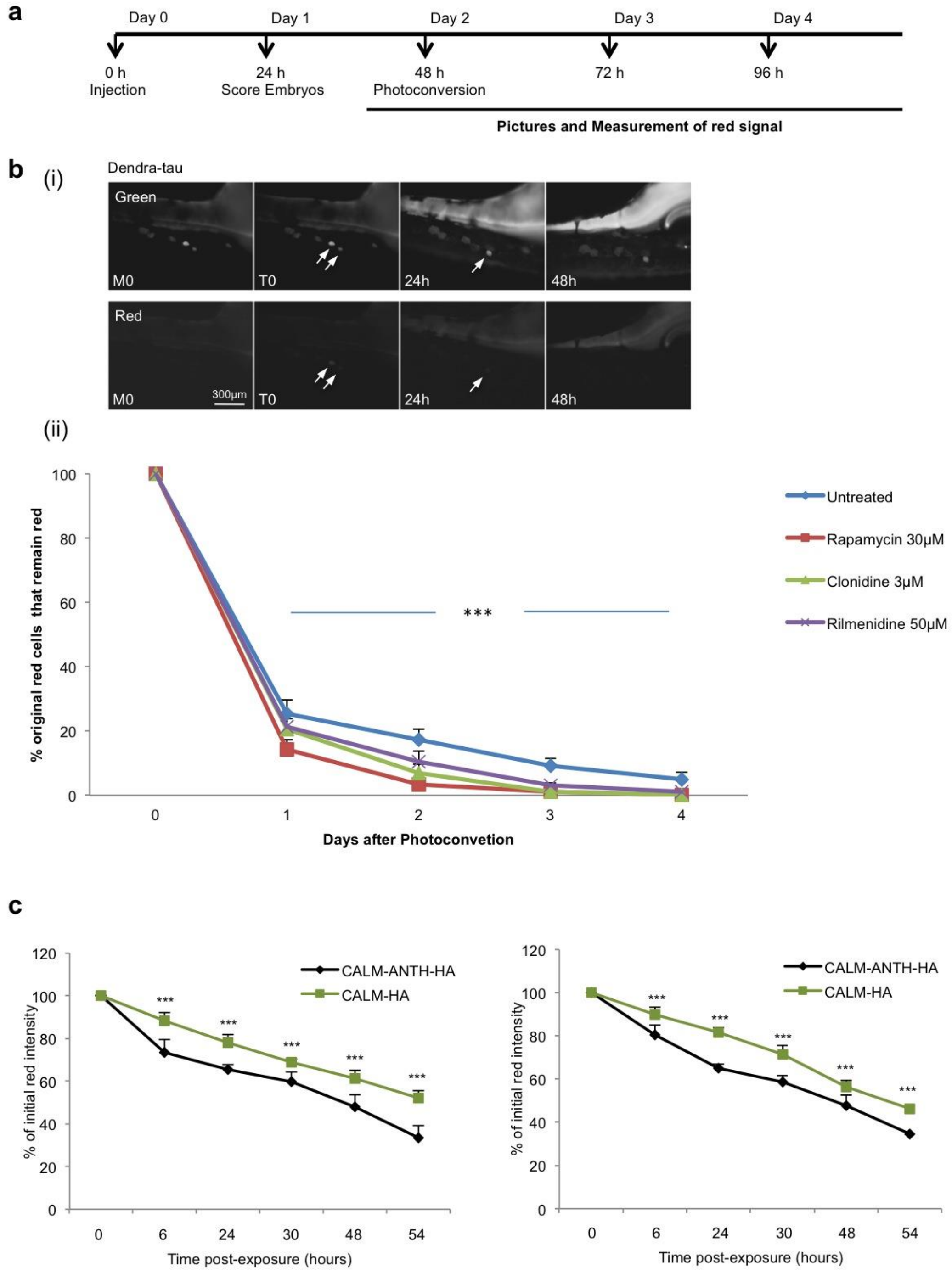

Supplementary Figure 6. CALM regulates tau clearance in zebrafish.
(a). Schematic representation of the Dendra-tau clearance experiment. Dendra-tau was injected into zebrafish embryos at the one cell stage. At 24 h.p.f., embryos were viewed using EGFP filter sets on a microscope to identify those with mosaic expression of Dendra-tau. Dendra fluorescent protein was photoconverted from green to red by exposure of whole larvae to 365 nm light at 48 h.p.f. Digital images were captured using fluorescent microscope, a digital camera and a capture software before and after photoconversion and at defined time points thereafter to monitor Dendra-tau clearance.

(bi). Representative images of larvae with mosaic expression of Dendra-tau taken prior to photoconversion (M0), immediately after photoconversion (T0) and at 24 h and 48 h after conversion (green fluorescence, top row; red fluorescence, bottom row).

(bii). Modulation of autophagy alters Dendra-tau clearance dynamics. The number of red fluorescent cells was counted daily and expressed as a percentage of the number of red fluorescent cells immediately after photoconversion and at 24 hours intervals thereafter. (i). The autophagy enhancing drugs rapamycin, clonidine and rilmenidine all significantly increased the rate of Dendra-tau clearance. ($n \geq 60$ cells, n=12 larvae per treatment group) (***: $p < 0.001$, One-way ANOVA). Error bars are mean +/- SD. Representative images of larvae with mosaic expression of Dendra-tau taken prior to photoconversion (M0), immediately after photoconversion (T0) and at 24 h and 48 h after conversion (green fluorescence, top row; red fluorescence, bottom row) are shown. Note that (i) and (ii) are distinct experiments.

(c). Quantification of Dendra-tau clearance in zebrafish larvae co-expressing CALM. Images of zebrafish larvae with mosaic expression of Dendra-tau and full-length CALM or Δ–ANTH CALM (CALM-ANTH-HA) taken immediately after photoconversion (0) and at 6 h, 24 h, 30 h, 48 h and 54 h after conversion were subjected to immunofluorescence analysis. The fluorescence intensity of individual cells was quantified and mean fluorescent intensity per treatment group ($n \geq 100$ cells, ≥9 larvae per treatment group) was plotted for each time point to calculate clearance of red Dendra-tau over time. Expression of full length CALM significantly delayed the clearance of Dendra-tau at all time points compared to Δ–ANTH CALM ($p<0.01$, One-way ANOVA). 2 independent experiments are presented. Error bars are mean +/- SD. ***: $p<0.01$.

Supplementary Figure 7: CALM regulates autophagy and tau toxicity in zebrafish

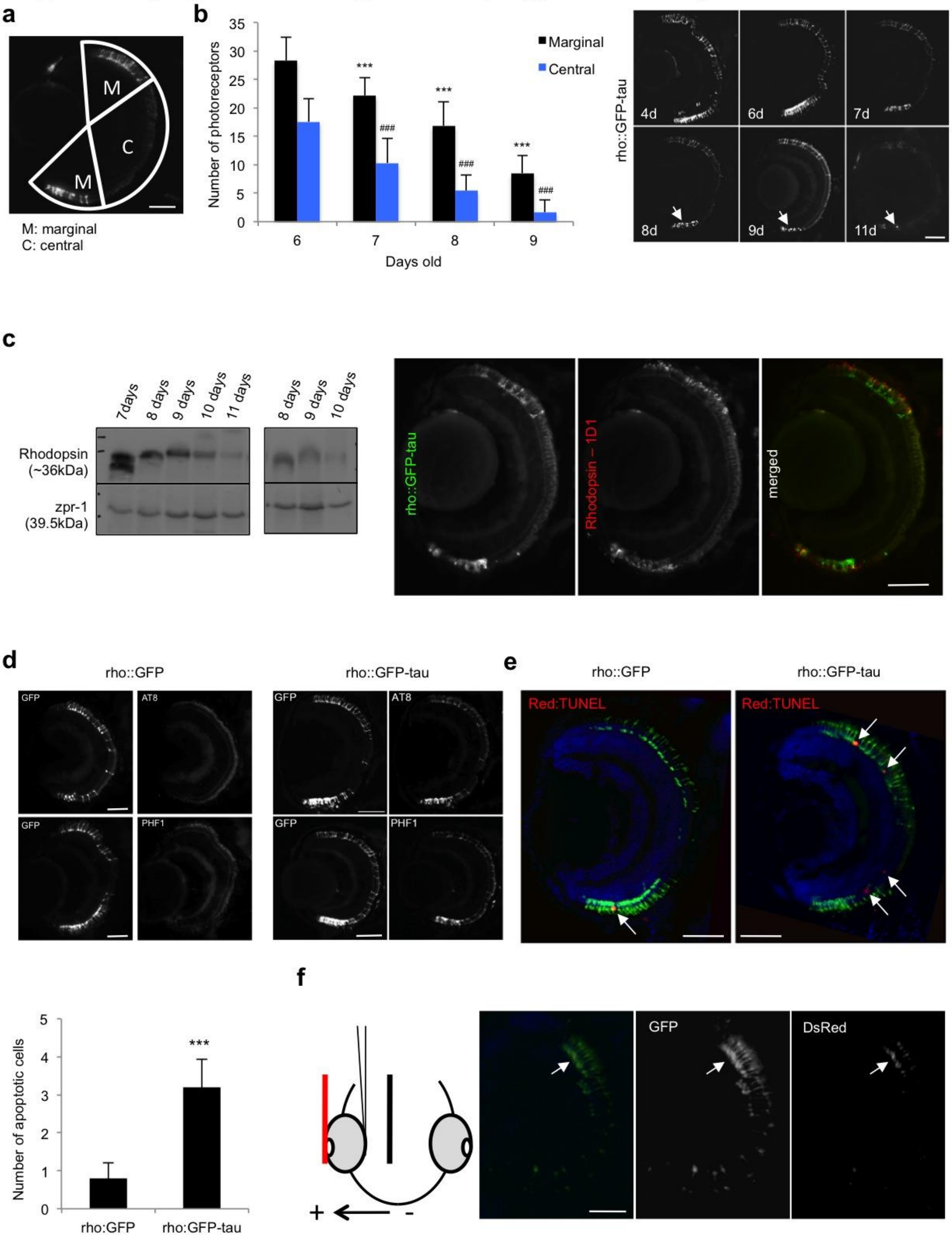

Supplementary Figure 7. CALM regulates autophagy and tau toxicity in zebrafish.

(a). Quantification of rod photoreceptor degeneration in rho::GFP-tau zebrafish. The retina was divided into the marginal (M) and central (C) regions and the total number of EGFP-positive rod photoreceptors was counted from sections through the optic nerve head of the retina of n=18 eyes (9 larvae) from 6 to 9 d.p.f. The mean number of photoreceptors was calculated for each time point. Scale bar, 50 μm.

(b). Tau-associated degeneration in a zebrafish transgenic model. Representative images of the retina of transgenic rho::GFP-tau zebrafish in the region of the optic

nerve head from 4 d.p.f. (days post-fertilization) to 11 d.p.f. Rod photoreceptors arise from the ventral-nasal portion of the retinal from 3 d.p.f. onwards. Strong GFP expression is in the ventral retina at 4 to 6 d.p.f. and in rods throughout the photoreceptor layer. By 7 d.p.f., degeneration of GFP-tau positive rods is observed in the ventral region and there is an absence of rods from the central regions of the retina. Further degeneration in the ventral region is observed at 9 d.p.f. and fluorescent aggregates are observed (arrows). By 11 d.p.f., almost all rod photoreceptors are lost and bright fluorescent aggregates are present in the ventral retina (arrows). Scale bar, 50 μm. Quantification of rod photoreceptor degeneration is shown: the retina was divided into the marginal and central regions and the total number of GFP-positive rod photoreceptors counted from sections through the optic nerve head of the retina ($n \geq 18$ eyes; 9 larvae per time point; error bars are mean +/- SD, ***: p<0.001 relative to marginal region at 6 d.p.f.; ###: p<0.001 relative to central region at 6 d.p.f., One-way ANOVA).

(c). Western blot analysis of rod photoreceptor degeneration in rho::GFP-tau fish. Rhodopsin protein (detected with the rhodopsin antibody Zpr3), the major component of the rod outer segment decreases dramatically from 7 d.p.f. to 11 d.p.f., whilst the cone photoreceptor marker (Zpr-1) remains unchanged, indicating a specific degeneration in tau-expressing rod photoreceptors. Rod photoreceptor degeneration in rho::GFP-tau fish can be quantified by visualisation of GFP-positive cells. Immunohistochemistry using the anti-zebrafish rhodopsin antibody, 1D1 [2] through the retina of rho::GFP-tau fish at 9 d.p.f. (shown on the right) demonstrates that rod degeneration occurs throughout the retina in rho::GFP-tau fish. Rhodopsin is observed in the rod outer segments and GFP is observed in the cell body of the same cell. All antibody-positive rod photoreceptors (red) are also GFP-positive (green), indicating that GFP is a reliable indicator of the presence/absence of rods and that loss of GFP-positive cells indicates degeneration rather than silencing of the transgene.

(d). Hyperphosphorylation of tau in rho::GFP-tau fish. At 6 d.p.f., AT8 antibody (a marker of phosphorylation at Ser202/Thr205) staining was observed in some GFP-tau positive rod photoreceptors. PHF1 antibody staining (Ser396/Ser404) was observed as early as 3 d.p.f. (not shown) and was evident in all GFP-tau positive rod photoreceptors at 6 d.p.f. Positive staining was also observed with AT270 (Thr181) from 3 d.p.f. onwards (not shown). No staining was observed in the retina of control rho::GFP fish. Scale bar, 50 μm.

(e). Quantification of cell death in the photoreceptors of rho::GFP-tau fish: Sections through the central retina demonstrated a marked increase in TUNEL-positive of nuclei in the photoreceptor of rho::GFP-tau fish compared to rho::GFP fish at 7 d.p.f. Quantification was performed by counting the total number of TUNEL-positive nuclei in the photoreceptor layer from the central retina and calculating mean values per group (***: p<0.01; two-tailed t-test; n=5 larvae per group).

(f). Electroporation of photoreceptor cell layer in larval zebrafish: Larvae at 4 d.p.f. were anaesthetized and embedded in 1% LMP agarose. Electrodes were positioned within the brain (negative) and touching the outer edge of the lens (positive) of the left eye and a microinjection capillary was inserted into the orbit. At 24 hours after electroporation, dsRed-positive cells were observed in the photoreceptor layer (co-localised with GFP-positive rods) and in the outer surface of the dorsal retina. Scale bar, 50 μm

Supplementary Figure 8: CALM expression exacerbates degeneration in rho::GFP-tau zebrafish

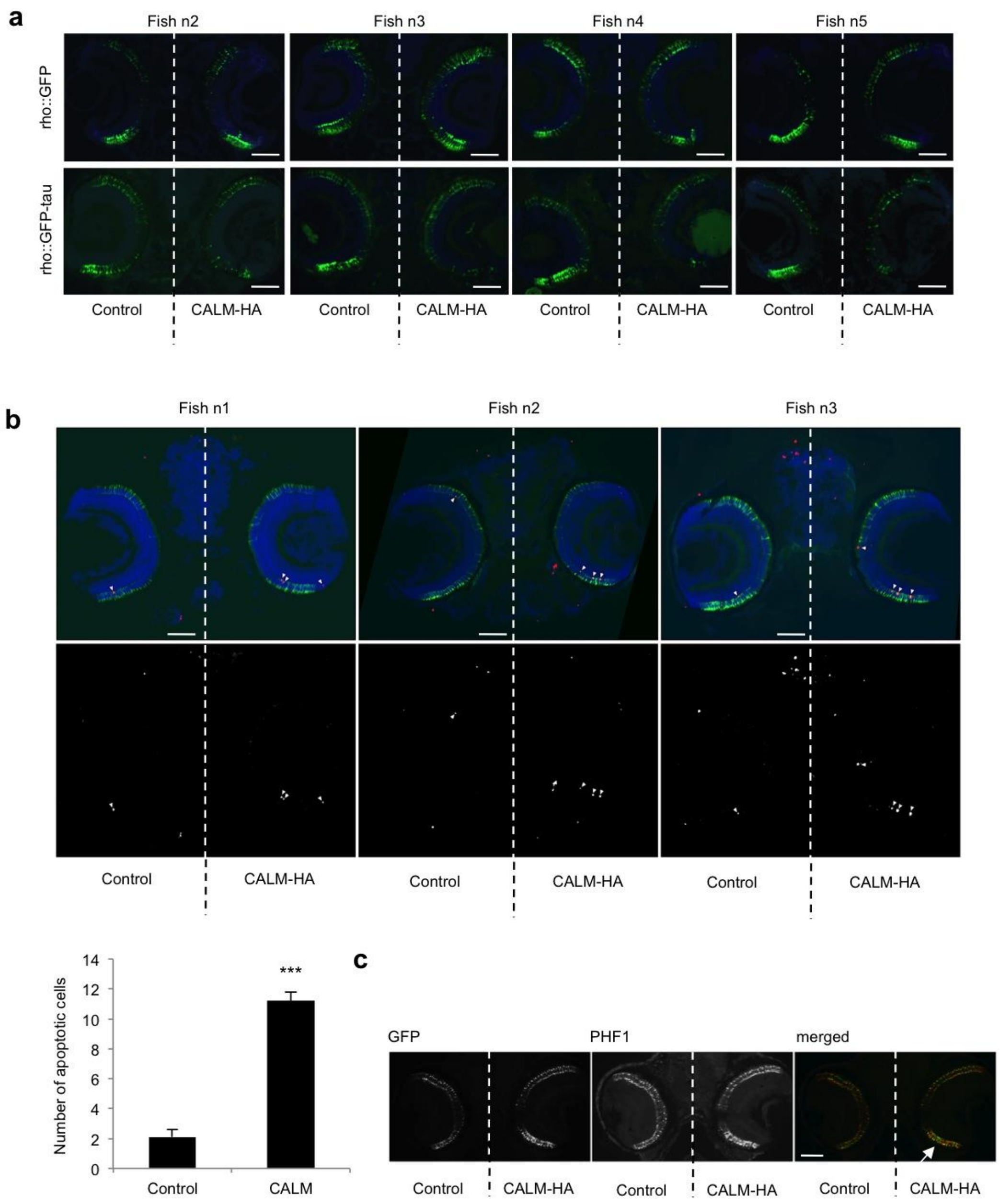

Supplementary Figure 8. CALM expression exacerbates degeneration in rho::GFP-tau zebrafish

(a). Further examples of fish from the experiment described in Figure 9B are presented. Scale bar, 50 μm.

(b). Cell death in the photoreceptors of rho::GFP-tau fish following CALM electroporation: Sections through the central retina of rho::GFP-tau fish demonstrated a marked increase in TUNEL-positive of nuclei in the photoreceptor after unilateral

CALM electroporation. Quantification was performed by counting the total number of TUNEL-positive nuclei in the photoreceptor layer from the central retina and calculating mean values per group (***: $p<0.01$; two-tailed *t*-test; n=9 larvae per group).

(c). Unilateral electroporation of CALM into the retina of rho::GFP-tau zebrafish resulted in a marked increase in phosphorylated tau (PHF1 antibody labelling, red) in the electroporated retina in the photoreceptor layer (PR) compared to the control side. Note, photoreceptor degeneration was blocked by treatment with the caspase inhibitor Z-VAD-FMK, hence an equal population of GFP-positive rod photoreceptors (green) are observed on control and CALM-electroporated sides.

Supplementary Figure 9: full blot images

1a

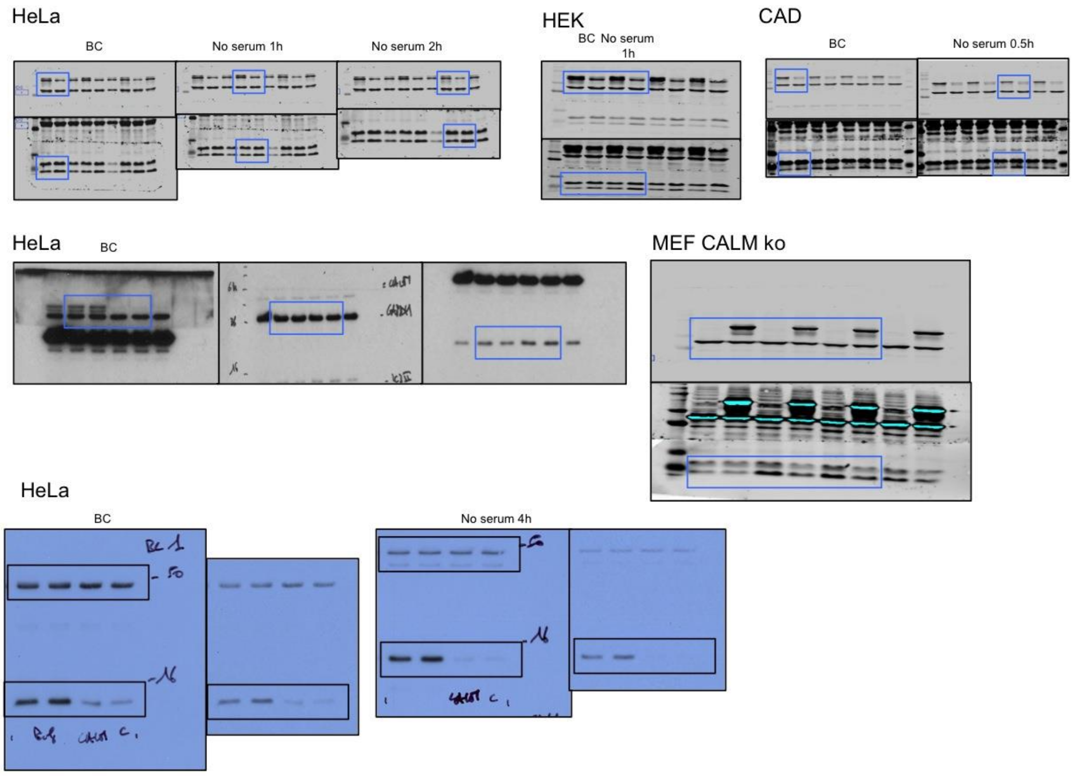

1b

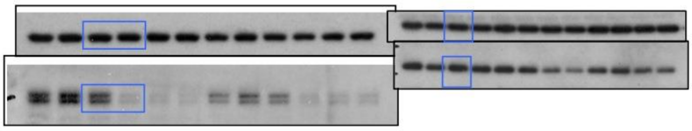

2b

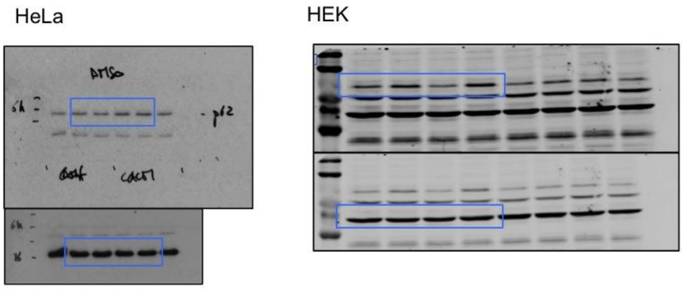

4a

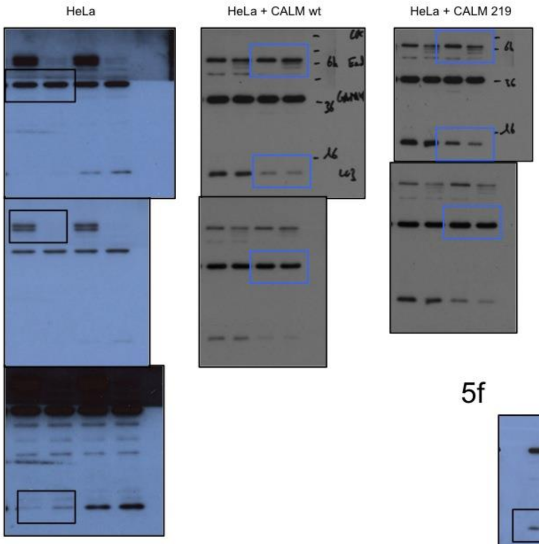

5b

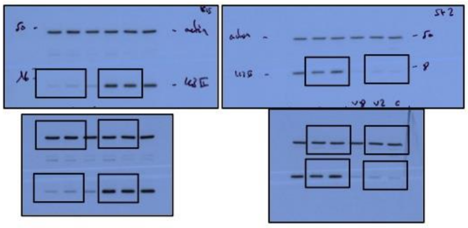

5f

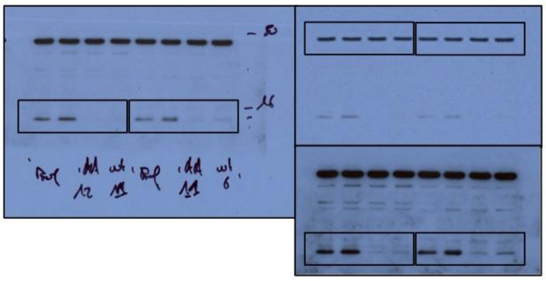

7a

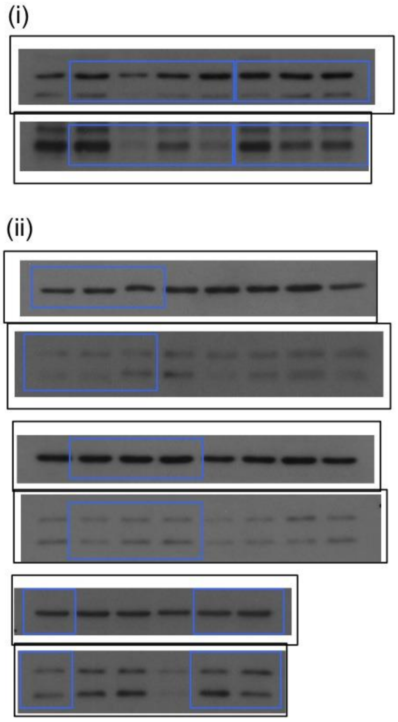

7b

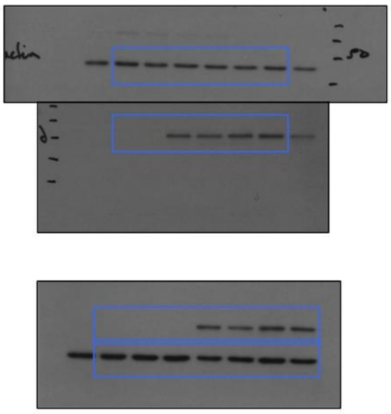

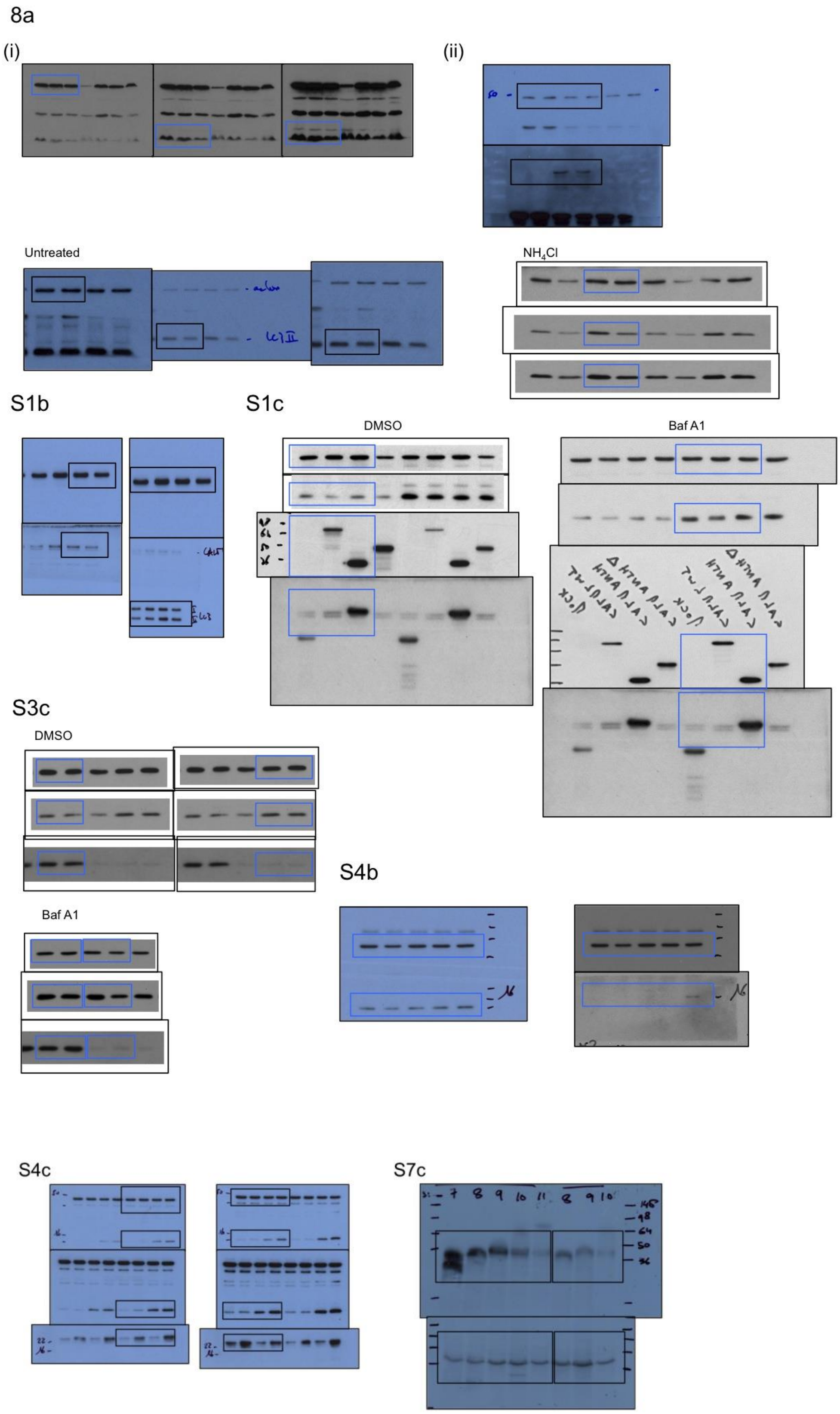

Supplementary Figure 9. Full blot images

Table 1: Effect of human tau expression in *Drosophila*

Phenotype (driver)	Effect on tau-WT
Lethality (tubulin-GAL4)	No effect
Eye (GMR-and ey-GAL4)	No effect
Locomotor activity (D42-GAL4)	No effect
Wing (Ccap-GAL4)	No effect
Pseudopupil (N-Syb-GAL4)	No effect

Table 1. Effect of human tau expression in *Drosophila*.
Human tau-WT was expressed in different tissues using specific drivers. We assessed the effects of tau expression on lethality using tubulin-GAL4 (P{tubP-GAL4}LL7; [3]), on eye phenotype using (P{GAL4-ninaE.GMR}12; [4]) and eyeless-GAL4 (ey-GAL4, P{GAL4-ey.H}3-8; [5]), on locomotor activity using D42-GAL4 (P{GawB}D42; [6]), on wing phenotype using Ccap-GAL4 (P{Ccap-GAL4.P}16 and P{Ccap-GAL4.P}9; [7]) and on the number of rhabdomeres using N-Syb-GAL4 1-2 (gift of Dr. Julie Simpson).

References

1. Wittmann, C.W. *et al.* Tauopathy in Drosophila: neurodegeneration without neurofibrillary tangles. *Science* **293**, 711-4 (2001).
2. Hyatt, G.A., Schmitt, E.A., Fadool, J.M. & Dowling, J.E. Retinoic acid alters photoreceptor development in vivo. *Proc Natl Acad Sci U S A* **93**, 13298-303 (1996).
3. Lee, T. & Luo, L. Mosaic analysis with a repressible cell marker for studies of gene function in neuronal morphogenesis. *Neuron* **22**, 451-61 (1999).
4. Freeman, M. Reiterative use of the EGF receptor triggers differentiation of all cell types in the Drosophila eye. *Cell* **87**, 651-60 (1996).
5. Hazelett, D.J., Bourouis, M., Walldorf, U. & Treisman, J.E. decapentaplegic and wingless are regulated by eyes absent and eyegone and interact to direct the pattern of retinal differentiation in the eye disc. *Development* **125**, 3741-51 (1998).
6. Parkes, T.L. *et al.* Extension of Drosophila lifespan by overexpression of human SOD1 in motorneurons. *Nat Genet* **19**, 171-4 (1998).
7. Park, D., Veenstra, J.A., Park, J.H. & Taghert, P.H. Mapping peptidergic cells in Drosophila: where DIMM fits in. *PLoS One* **3**, e1896 (2008).
